# MDR1A deficiency restrains tumor growth in murine colitis-associated carcinogenesis

**DOI:** 10.1371/journal.pone.0180834

**Published:** 2017-07-07

**Authors:** Eva Maria Hennenberg, Annette Eyking, Henning Reis, Elke Cario

**Affiliations:** 1 Experimental Gastroenterology, Department of Gastroenterology and Hepatology, University Hospital Essen, Essen, Germany; 2 Medical School, University of Duisburg-Essen, Essen, Germany; 3 Institute of Pathology, University Hospital Essen, Essen, Germany; Duke University, UNITED STATES

## Abstract

Patients with Ulcerative Colitis (UC) have an increased risk to develop colitis-associated colorectal cancer (CAC). Here, we found that protein expression of ABCB1 (*ATP Binding Cassette Subfamily B Member 1*) / MDR1 (*multidrug resistance 1*) was diminished in the intestinal mucosa of patients with active UC with or without CAC, but not in non-UC patients with sporadic colon cancer. We investigated the consequences of ABCB1/MDR1 loss-of-function in a common murine model for CAC (AOM/DSS). Mice deficient in MDR1A (MDR1A KO) showed enhanced intratumoral inflammation and cellular damage, which were associated with reduced colonic tumor size and decreased degree of dysplasia, when compared to wild-type (WT). Increased cell injury correlated with reduced capacity for growth of MDR1A KO tumor spheroids cultured ex-vivo. Gene expression analysis by microarray demonstrated that MDR1A deficiency shaped the inflammatory response towards an anti-tumorigenic microenvironment by downregulating genes known to be important mediators of cancer progression (PTGS2 (COX2), EREG, IL-11). MDR1A KO tumors showed increased gene expression of TNFSF10 (TRAIL), a known inducer of cancer cell death, and CCL12, a strong trigger of B cell chemotaxis. Abundant B220+ B lymphocyte infiltrates with interspersed CD138+ plasma cells were recruited to the MDR1A KO tumor microenvironment, concomitant with high levels of immunoglobulin light chain genes. In contrast, MDR1A deficiency in RAG2 KO mice that lack both B and T cells aggravated colonic tumor progression. MDR1A KO CD19+ B cells, but not WT CD19+ B cells, suppressed growth of colonic tumor-derived spheroids from AOM/DSS-WT mice in an ex-vivo co-culture system, implying that B-cell regulated immune responses contributed to delayed tumor development in MDR1A deficiency. In conclusion, we provide first evidence that loss of ABCB1/MDR1 function may represent an essential tumor-suppressive host defense mechanism in CAC.

## Introduction

Patients with long-term Ulcerative Colitis (UC) have an increased risk of developing colitis-associated colorectal cancer (CAC) [1]. Initial extent [2] and duration [3] of chronic inflammation are recognized to be among the key risk factors. DNA damage and methylation caused by oxidative stress in chronic inflammation drive tumor development and progression [4]. Microbiome-induced alterations in innate immune signaling may contribute to the development of intestinal inflammation and associated colon cancer in the susceptible individual [5]. CAC appears to be distinct from sporadic colorectal cancer (CRC), both in pathophysiological and clinical features. It usually affects individuals at a younger age than the general population and demonstrates a more proximal and multifocal distribution in the colon. CAC progresses to invasive adenocarcinoma from flat and nonpolypoid dysplasia more frequently and rapidly than CRC [6]. Some differences in the spectrum of genomic alterations were recently found in CAC compared with CRC [7]. Allelic deletion of the p53 tumor suppressor gene occurs early in the process of tumorigenesis [8] and more frequent in CAC, while mutations in APC are relatively rare [7]. But the mechanisms which may modulate tumor progression in CAC remain to be elucidated.

P-glycoprotein (p-gp), which is encoded by ABCB1 (*ATP Binding Cassette Subfamily B Member 1*) / MDR1 (*multidrug resistance 1*), functions as an ATP-dependent efflux transporter pump of xenobiotics (including metabolic products, toxins and drugs) at the intestinal mucosal barrier [9]. Numerous exogenous and endogenous factors may modulate ABCB1/MDR1 p-gp expression and function, including oxidative or inflammatory stress, microbial or dietary antigens, environmental or drug components as well as innate or adaptive immune responses [9]. Several lines of evidence suggest a potential role of ABCB1/MDR1 in both intestinal inflammation and tumorigenesis in the gastrointestinal tract. The ABCB1/MDR1 promoter region contains multiple transcription factor-binding sequences which link to pathways that trigger intestinal inflammation and tumorigenesis, such as TCF/LEF [10], RAS, p53 [11] and HIF-1α [12]. Gene expression of ABCB1/MDR1 has been shown to be reduced in active UC [13] and early CRC [14]. Mice deficient in MDR1A [15] develop spontaneously chronic colitis that resembles human UC [16] with continuous inflammation of the entire colon and mucosal thickening with crypt abscesses and distortion. Deletion of TLR2 causes fulminant exacerbation of pancolitis through commensally-induced pyroptosis in the context of MDR1A deficiency [17]. When dually infected with *Helicobacter bilis* and *H*. *hepaticus*, MDR1A knockout (KO) mice are prone to develop high-grade crypt dysplasia, including invasive adenocarcinoma, in bacteria-triggered colitis [18]. In contrast, P-gp-deficient *APC*^Min/+^ mice develop fewer polyps in the non-inflamed small intestine than wild-type (WT) [19]. The C3435T and intron 3 (G-rs3789243-A) polymorphisms of the human ABCB1/MDR1 gene have been associated with increased risks for UC [20–22] or CRC [23–26] in some populations, but not all [24, 27, 28]. However, the precise role of ABCB1/MDR1 in CAC pathogenesis has not been delineated yet. Here, we found that the expression of ABCB1/MDR1 protein was lost in the inflamed mucosa of UC patients with or without colon cancer. Using a murine model of CAC, we provide, to the best of our knowledge, first functional evidence that MDR1A deficiency plays a protective role against tumor progression of CAC.

## Materials and methods

### Antibodies and reagents

A detailed list of all antibodies is provided in S1 Table. All other reagents were obtained from Thermo Fisher Scientific (Waltham, MA) or Sigma-Aldrich (St. Louis, MI), unless otherwise specified.

### Human colonic specimens

We performed a retrospective, single-center cohort study among patients with a diagnosis of 1. active Ulcerative Colitis with colitis-associated colorectal carcinoma (CAC), 2. active Ulcerative Colitis without colorectal cancer (UC), or 3. sporadic colorectal cancer without Ulcerative Colitis (CRC), using formalin-fixed paraffin-embedded (FFPE) tissue blocks from surgical resection specimens archived from 2001 to 2016 at the Institute of Pathology, University Hospital Essen, Essen, Germany. For each tumor case (CAC or CRC), only matched adjacent non-neoplastic tissue (R_0_) and tumor block pairs were included. All human tissue material was reviewed by a board-certified pathologist (H.R.). S2 Table shows the histopathological features of the 54 patients. Of note, our patients with CAC showed a less advanced tumor stage distribution with rarer metastasis than patients with CRC at the time of diagnosis, because all UC patients underwent increased endoscopic surveillance and tumor lesions were therefore detected at an earlier stage than in the non-UC population, as previously described [29]. This study (anonymous analysis of historical collection) abided by the Declaration of Helsinki and was approved by the local Ethics Committee of the Medical Faculty of the University Duisburg-Essen, Germany (“Ethik-Kommission der Medizinischen Fakultät der Universität Duisburg-Essen”; permit #13-5602-BO).

### Animals

WT and parental MDR1A KO (originally developed [15] by Dr. Alfred Schinkel, The Netherlands Cancer Institute, Amsterdam, Netherlands) mice [all FVB/N; >F7] were obtained from Taconic Farms (Germantown, NY) under crossbreeding agreement [17]. Recombination activating gene 2-deficient mice (RAG2 KO) on the FVB/N background [30] were kindly provided by Dr. Thomas Kupper (Harvard Medical School and The Brigham and Women’s Hospital Inc., Boston, MA), rederived by Charles River (Lyon, France) and backcrossed onto MDR1A KO [FVB/N] to generate double-KO (dKO) homozygotes (MDR1A/RAG2 dKO). Mice were confirmed to be the desired genotype via standard genotyping techniques. All mice were bred and housed in the same temperature- and humidity-controlled room on a 12-h light-dark cycle under strict specific-pathogen-free conditions (Central Animal Facility, University Hospital Essen). The animals were provided with autoclaved tap water and autoclaved standard laboratory chow (ssniff M-Z, ssniff Spezialdiäten, Soest, Germany) *ad libitum*. Extensive animal health monitoring (criteria of the Federation for Laboratory Animal Science Associations (FELASA)) was conducted routinely on sentinels (Gesellschaft für innovative Mikroökologie, Wildenbruch, Germany) and representative mice from this room and no pathogens were detected. For studies, only age-matched male mice were used. Prior to use in experiments, mice were placed individually into a fresh filter-top cage and allowed to acclimatize for at least 3 days. Protocols were in compliance with German law for use of live animals and regulations of the Society for Laboratory Animal Science (GV-SOLAS) and the FELASA. The study was prospectively reviewed and approved by the local animal protection officer at the University Hospital Essen and the responsible district government (North Rhine-Westphalia State Agency for Nature, Environment and Consumer Protection, Recklinghausen, Germany; permit number G1377/13). All mice were sacrificed by cervical dislocation under isoflurane anesthesia. All efforts were made to minimize animal suffering and to reduce the number of animals used.

### Murine model of CAC

We used the common azoxymethane (AOM) / dextran sulfate sodium (DSS)–model to study the development of CAC in MDR1A-deficient mice [31]. AOM (lots# SLBG5125V, SLBN5975V) was administered once (10mg/kg body weight) to 6-week-old male mice, followed by treatments with 2.5% DSS (mol wt, 36,000–50,000; lot# M5164; MP Biomedicals, Santa Ana, CA) for 7 days in drinking water starting on day 0, 21 and 42, respectively. The planned experimental endpoints were week 12 (WT, MDR1A KO) or week 20 (RAG2 KO, MDR1A/RAG2 dKO). At that time the entire colon and rectum were excised, cut longitudinally, and rinsed in ice-cold Hank’s Balanced Salt Solution. During gross examination, tumors were macroscopically visualized by 1% alcian blue staining, counted and measured (longest diameter) [32]. During the study, health and behaviour of each mouse were closely monitored and evaluated at least once per day by adequately trained and experienced animal research personnel at the University Hospital Essen. In order to prevent unnecessary animal pain and distress, humane endpoints were established prior to the start of the study, in order to decide when to terminate the experiment. These humane endpoints included body weight loss (>20%), alterations in external physical appearance (hunched posture, signs of dehydration) and behavioural changes (apathy, laboured respiration). Mice were to be immediately sacrificed when any of these humane endpoints were reached.

### Colonic tumor spheroid culture

The supportive cell line L-WRN was kindly provided by Dr. Thaddeus Stappenbeck (Washington University School of Medicine, St. Louis, MO) and grown in high-glucose DMEM supplemented with 10% FCS (lot# RYF35911), 100U/ml penicillin and 100μg/ml streptomycin. Conditioned media was collected using advanced DMEM/F12 medium supplemented with 20% FCS, 100U/ml penicillin, 100μg/ml streptomycin, and 2mM L-glutamine, as previously described [33], and 1% antibiotic-antimycotic and 0.1% gentamycin were added. Colonic tumor spheroids were also processed and cultured as previously described [33]. In brief, the colonic tumor tissue was excised from WT or MDR1A KO mice after AOM/DSS treatment at the experimental endpoints and then cut into small pieces and digested with collagenase I (2mg/ml) for 1h at 37°C with vigorous pipetting every 5–10 mins. After passage through a cell strainer (BD Biosciences, Bedford, MA) and centrifugation (200g, 4°C for 5 mins), the pellet was suspended with cold Matrigel (lots #6074313, #6102305; Corning, Corning, NY) and 15μl of this mixture was added to each well of a 24-well plate (for culture) or a 6-well plate (for experiments). After polymerization (37°C, 10 mins), conditioned media from L-WRN cells (500μl/24-well format; 2ml/6-well format) containing 10μM SB431542 and 10μM Y-27632 (Selleckchem, Houston, TX) was overlaid. Tumor spheroids [34] were passaged by mechanical dissociation and photographed using EVOS fl (Advanced Microscopy Group, Bothell, WA) at the Imaging Centre Essen, University Hospital Essen (IMCES).

### Murine cell isolation and culture

Bone marrow cells and splenocytes from untreated, age- and gender-matched WT or MDR1A KO mice were isolated and resuspended in ice-cold 2% FCS (PBS). Colonic lamina propria mononuclear cells were isolated as previously described [17]. In brief, colons were cut open longitudinally, washed with ice-cold 2% FCS (PBS), and digested with dispase (2mg/ml) at 37°C for 50 mins in complete media (RPMI 1640 media supplemented with 4% FCS, 2.5% HEPES and 1% antibiotic-antimycotic) to remove the epithelial compartment. Samples were then minced into small pieces and further digested with collagenase II (1.5mg/ml) and dispase (1.0mg/ml) for 50 mins at 37°C. For B cell purification, splenocytes were passed through a 70μm-cell strainer (BD Biosciences) and purified using the EasySep™ Mouse CD19 Positive Selection Kit II (STEMCELL Technologies, Vancouver, Canada). Purity of B cells was confirmed by flow cytometry analysis (B220+ ≥ 93%). WT or MDR1A KO–CD19+ B cells were maintained in IMDM supplemented with 10% FCS, 100U/ml penicillin, 100μg/ml streptomycin and 4ng/ml recombinant BAFF (Biolegend, San Diego, CA), with > 80% B220+ and > 80% CXCR4+ cells after 24h-culture. Freshly isolated CD19+ B cells (4x10^6^ cells/2 ml) were co-cultured with pre-plated WT tumor spheroids for 24h in a Transwell® co-culture system (0.4μm-insert (Corning); 6-well format), as depicted in S1 Fig.

### Flow cytometry analysis

After washing and incubation with Mouse BD Fc Block™ (purified rat anti-mouse CD16/CD32 (BD Pharmingen)), cells were analyzed using a BD FACS Canto™ II (BD FACSDiva™ Software version 8.0.1) after staining with rat anti-B220-PE-CY7, anti-CD19-PerCP-CY5.5, anti-CD43-APC, anti-CD138-APC, anti-CXCR4-PE, anti-IgD-PE and/or anti-IgM-FITC (S1 Table). Cells were fixed in freshly prepared 0.5% paraformaldehyde (Electron Microscopy Sciences, Hartfield, PA) at 4°C o/n and analyzed the next day. All Abs and appropriate isotype IgG controls were purchased from BD Biosciences or BioLegend. Dead cells were excluded by Fixable Viability Dye eFluor®450 and compensation for spectral overlap was set using BD CompBeads Set (BD Pharmingen). Flow cytometry data were analyzed using FlowJo software for PC (version 7.6.5; Tree Star, Ashland, OR).

### Histopathology and immunohistochemistry

Histopathological analysis was performed by hematoxylin-eosin (H&E) staining (Carl Roth, Karlsruhe, Germany) following standard protocols. Murine neoplastic lesions were evaluated based on the WHO classification of tumors of the digestive system [35] and scored (0—no dysplasia; 1 –low-grade intraepithelial neoplasia; 2 –high-grade intraepithelial neoplasia; 3 –adenocarcinoma) by the pathologist (H.R.) blinded to their genetic and clinical information. The degree of inflammation in murine tumor areas (*inflammatory score I*) was graded using a simple scoring system for absence or presence (0—*no*; 1—*yes*) of 1. erosion/ulceration, 2. crypt abscesses and 3. markedly increased quantity of inflammatory cell infiltration. The degree of inflammation in murine non-tumor areas (*inflammatory score II*) was graded as previously described [17]. For immunohistochemistry, FFPE cross-sections (5μm) were stained according to S3 Table, incubated with a HRP-conjugated detection reagent for 30 mins and antibody binding was visualized using SignalStain® DAB Substrate Kit (Cell Signaling Technology, Danvers, MA). Sections were counterstained using Vector® Hematoxylin QS (Vector Laboratories, Burlingame, CA).

### Immunofluorescence

Frozen sections of tissues were cut (7μm), mounted on Superfrost Plus Gold slides and fixed with 3% paraformaldehyde for 15 mins at RT. Sections were blocked with 5% normal goat serum (Vector Laboratories)/0.3% TX-100/PBS for 60 mins at RT and incubated with anti-p-histone H2A.X (1:100) in 1% normal goat serum/0.3% TX-100/PBS o/n at 4°C. AlexaFluor® 488-conjugated goat-anti-rabbit antibody (Cell Signaling; cat. #4412) was used as secondary antibody (1:50, 60 mins, RT). After mounting with Vectashield Mounting Medium containing propidium iodide (Vector Laboratories), immunofluorescent sections were assessed by confocal laser microscopy (Axiovert 100M with laser scan head LSM 510; Carl Zeiss, Jena, Germany). The multitrack option and sequential scanning for each channel were used to eliminate any cross talk of the chromophores. As positive control for p-histone H2A.X-labelled cells, frozen jejunal sections from TLR2 KO mice treated with methotrexate were used [36].

### Image analysis

High-resolution images were captured using the Aperio ScanScope system (Leica Biosystems, Nussloch, Germany) and visualized using ImageScope software (version 11.2.0.780, Leica Biosystems). In all experiments, at least 4 individual sites of image capture were chosen randomly for each sample. Morphological results were considered significant only if at least 70% of the scanned sections per field exhibited the observed effect. To quantify the p-histone H2A.X- or p-histone H3-positively labelled cells, 5–6 randomly chosen vision fields of each slide at an original magnification of 20x or 40x were counted blinded [37] using ImageScope, as indicated. Tumor spheroid areas [mm^2^] were measured using an ImageJ (version 1.46r, National Institutes of Health, Bethesda, MD) automated routine.

### RNA extraction

Total RNA from colonic tumors was extracted (RNeasy Micro Kit (Qiagen, Hilden, Germany) or RiboPure plus RNeasy Mini Kit (Qiagen)) including the RNase-free DNase digestion step. To synthesize cDNA, the QuantiTect® Reverse Transcription Kit (Qiagen) was used. FFPE tissue samples were cut into 10–12μm-thick sections on a microtome with a disposable blade (in part after macrodissection to enrich for region-of-interest content, as needed). RNA was extracted from 3 to 5 sequential sections from the same paraffin block using the RNeasy FFPE Kit with the deparaffinization solution (Qiagen), as previously described [29].

### Microarray analysis

All RNA samples (one tumor per individual mouse; *n* = 4 per group) were analyzed independently and further processed at the “KFB Center of Excellence for Fluorescent Bioanalytics” (Regensburg, Germany), where hybridization was performed using GeneChip® Mouse Gene 2.0 ST arrays (Affymetrix), according to the manufacturer’s instructions. Summarized probe set signals in log2 scale were calculated by using the RMA (Robust Multichip Average) algorithm with the Affymetrix GeneChip® Expression Console v1.4 Software. Data have been deposited in GEO (GSE94960) https://www.ncbi.nlm.nih.gov/geo/query/acc.cgi?acc=GSE94960. Data were further classified through Ingenuity Pathways Analysis (IPA, Ingenuity Systems®, www.ingenuity.com).

### Realtime qPCR expression analysis

Realtime qPCR analysis was performed at least in duplicate using the QuantiTect SYBR® Green PCR Kit (Qiagen) on the “Mastercycler ep realplex^2^” (Eppendorf, Hamburg, Germany) real-time amplification system. QuantiTect Primer Assays (Qiagen) were used as the gene-specific primer pairs. For FFPE-derived RNA samples, optimized gene-specific human primers were designed using Primer BLAST software: ABCB1: 5’-ATA AAA GAG AGG TGC AAC GGA AGC C-3’ and 5’-TCG AGA AAC TGC GAA ACA GGT TGA-3’; GAPDH: 5'-CCC ATC ACC ATC TTC CAG GAG CGA-3' and 5'-GCC AGC ATC GCC CCA CTT GA-3' (Eurofins MWG, Ebersberg, Germany). Copy numbers of individual transcripts were related to GAPDH as endogenous control (x/100,000 copies GAPDH) and normalized as indicated.

### Statistical analysis

The unpaired *t* test was used to calculate differences between means (GraphPad Prism version 5.04; GraphPad Software, La Jolla, CA). All tests were two-tailed, and *p* values of < 0.05 were considered as significant. All data are expressed as means ± SEM. Microarray probe sets with a fold change ≥ 2.0 and a *p* value of < 0.05 were considered as significantly regulated.

## Results

### Analysis of ABCB1 gene and protein expression in human CAC

**Fig 1:**
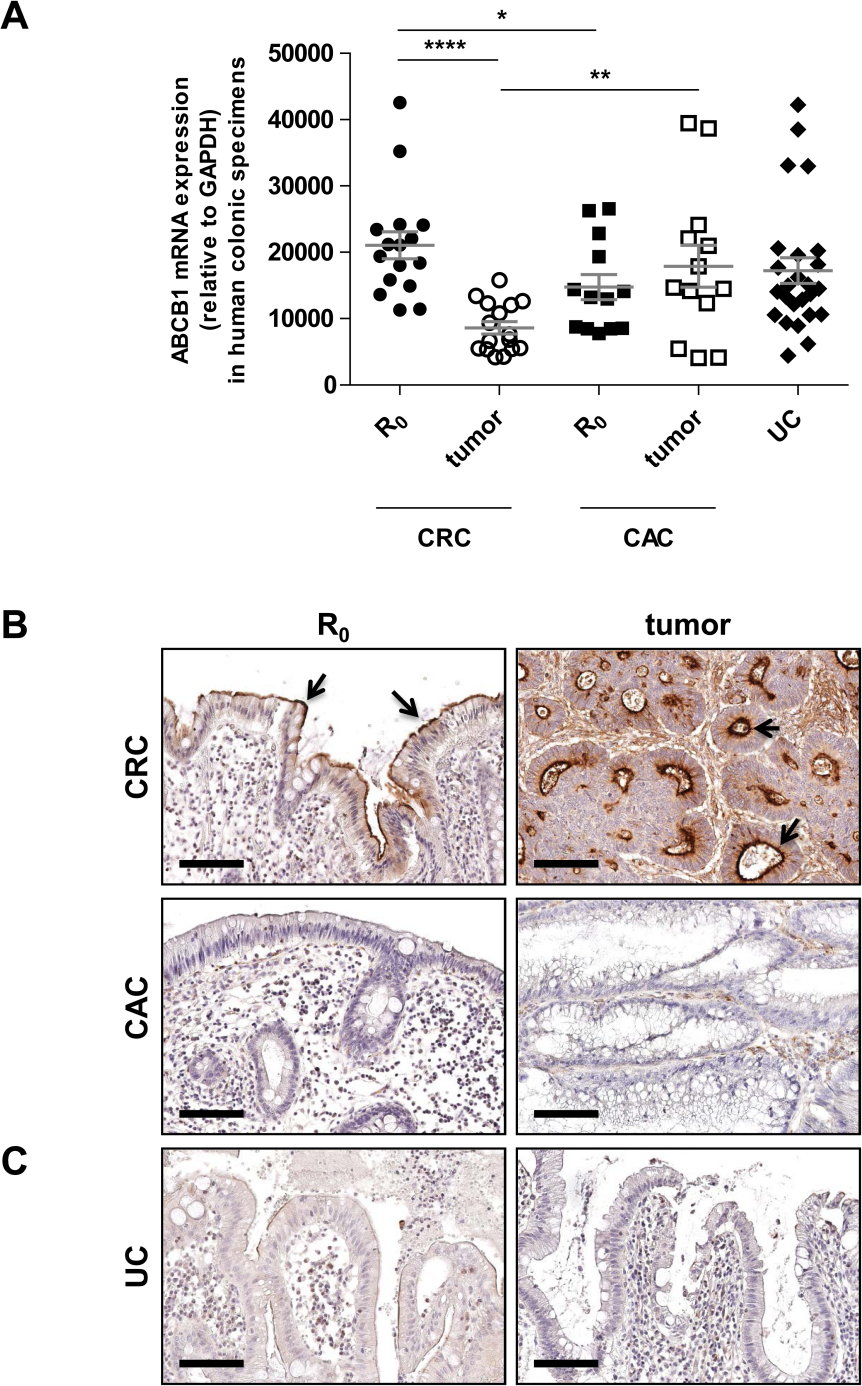
ABCB1 mRNA/protein expression in human CAC patient samples. (A) Expression of ABCB1 mRNA is highly variable in paired CAC tumor tissues and corresponding R_0_ margins as well as in active UC tissues without colorectal cancer, as determined by qPCR. In contrast, human sporadic CRC tumor areas express low levels of ABCB1 mRNA compared to matched R_0_ margins. Data are presented as means ± SEM (CAC: *n* = 13; CRC: *n* = 16; UC: *n* = 25): *p < 0.05, **p < 0.01, ****p < 0.0001. (B) ABCB1 protein expression is minimal in the inflamed, but tumor-free R_0_ margin of CAC tissues and remains unchanged in tumor areas, as determined by immunohistochemistry. In contrast, constitutive ABCB1 protein is predominantly expressed on surface IEC at normal margins of CRC and corresponding tumor cells. Results are shown for a representative patient of each tumor entity (total: CAC: *n* = 12; CRC: *n* = 10). (C) ABCB1 protein expression is expressed at low levels on surface IEC in active UC without colorectal cancer. Results are shown for two different UC patients (total: UC: *n* = 18). (B+C) *Black arrows* indicate examples of positive cells. Scale bar: 100μm.

First, we assessed the mRNA and protein expression patterns of ABCB1 in human specimens of CAC, in comparison to UC without colorectal cancer and CRC without UC, respectively. As shown in Fig 1A, ABCB1 mRNA expression levels were highly variable in UC-related colonic specimens, regardless of the histological diagnosis (active inflammation with or without tumor disease). In contrast, expression of ABCB1 mRNA was consistently decreased in tumor lesions of CRC, when compared to adjacent normal, non-inflamed colonic mucosa (R_0_).

Epithelial or lamina propria mononuclear expression of ABCB1 protein was barely detectable across tumor-free and tumor tissues from CAC patients (Fig 1B). 10 out of 12 CAC tumor samples showed completely lost or only very weak staining of ABCB1 protein expression. No ABCB1 protein expression was observed in 6 out of 12 colonic specimens of inflamed but tumor-free margins from CAC patients. Staining for ABCB1 was also diminished in inflamed mucosae in most tissue samples (15 out of 18) from active UC patients without colon cancer (Fig 1C). In contrast, in non-inflamed, normal margins of CRC (Fig 1B), intense staining of ABCB1 protein was consistently present at the apical pole of intestinal epithelial cells (IEC). In all tumor samples from CRC patients, abundant ABCB1 protein was present on cell surfaces and cytoplasm of IEC. Lamina propria mononuclear cells also stained positively for ABCB1 in tumor specimens from CRC patients, while scattered lamina propria mononuclear cells showed weak ABCB1 protein expression in healthy margins of CRC specimens. These data indicate that active UC–with or without colon cancer–may be associated with significant loss of ABCB1 protein expression in the intestinal mucosa, when compared to normal controls or CRC.

### MDR1A deficiency attenuates tumor progression in murine CAC

**Fig 2:**
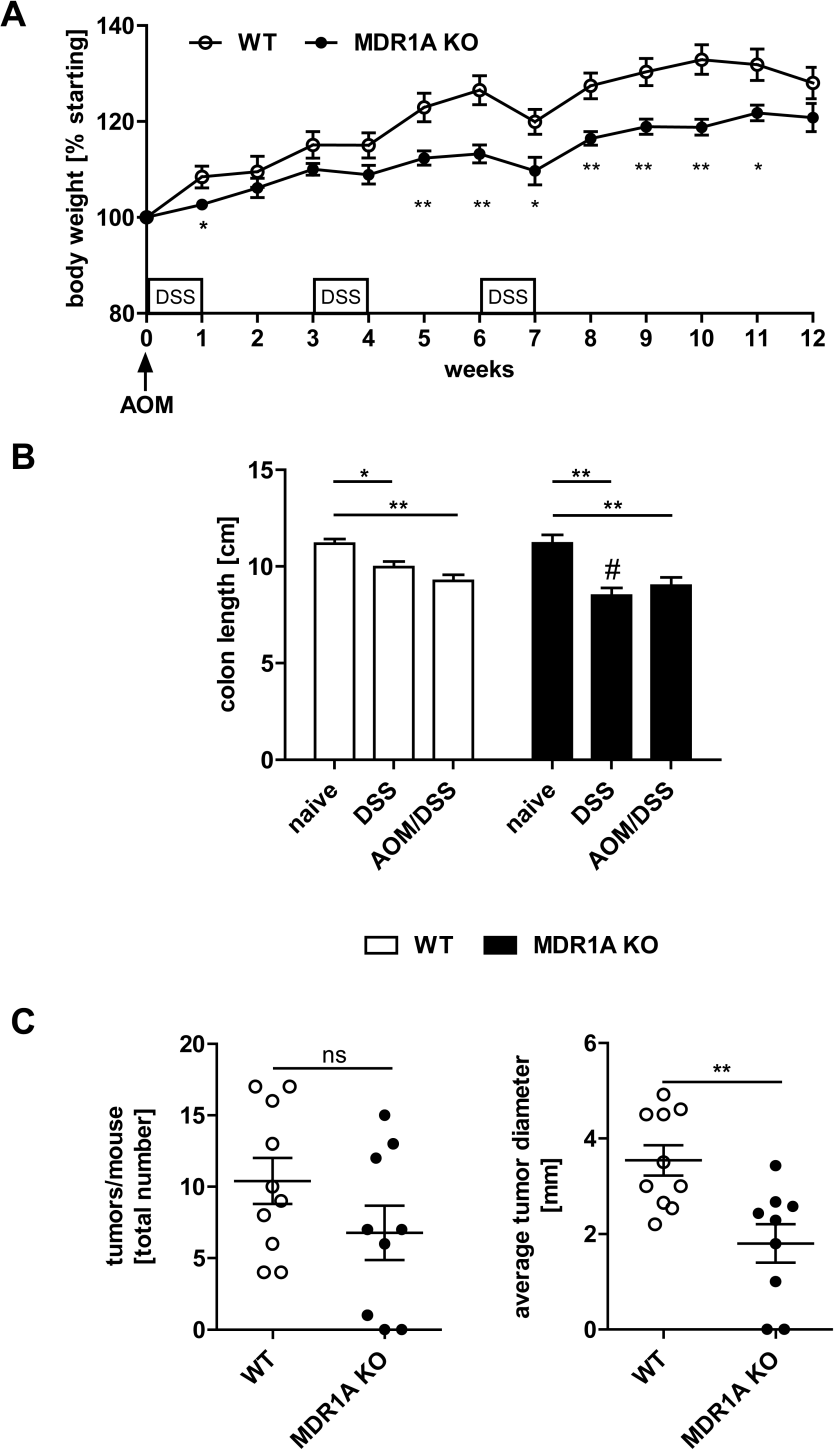
MDR1A deficiency suppresses colonic tumor growth in a mouse model of CAC. WT and MDR1A KO mice were intraperitoneally injected with AOM (10mg/kg body weight) and subjected to 3 cycles of DSS treatment (1 cycle representing 7 days of 2.5% DSS followed by 14 days of H_2_O). (A) Percent changes in body weight during the CAC protocol are shown. In week 12, (B) colon length was measured, and (C) macroscopic tumors in colons were counted and diameters were measured, as described in *Materials and Methods*. Data pooled from at least two independent experiments are shown (*n* = 9–10 mice/genotype). (A-C) Data represent means ± SEM. *p < 0.05, **p < 0.01, ***p < 0.001, ns: not significant. ((B) #: DSS-MDR1A KO vs. DSS-WT: **p < 0.01).

Second, we aimed to determine the functional effects of MDR1A deficiency on inflammation-associated tumorigenesis using a common mouse model of CAC in-vivo. As previously described [17, 38], our MDR1A KO mice generally show only mild signs of colonic inflammation and some remain even disease-free, presumably due to the superior cleanliness of our animal facility. To ensure penetrance of colitis, we therefore employed the chemical colitogen DSS. WT and MDR1A KO mice were intraperitoneally injected with the procarcinogen AOM followed by 3 cycles of 2.5% DSS administration. 25% (3 of 12) of the AOM/DSS-MDR1A KO mice did not reach the experimental endpoint and had to be sacrificed early (days 10, 55 and 68) versus 9% (1 of 11) of the AOM/DSS-WT (day 48), in all cases due to body weight loss > 20%. In general, the AOM/DSS—treatment caused more body weight loss in MDR1A KO mice (Fig 2A). Colon length, a marker of inflammation [39], was significantly shortened in all DSS-treated mice, regardless of genotype (Fig 2B), but slightly more decreased in DSS-MDR1A KO than DSS-WT. WT and MDR1A KO mice developed tumors in the middle to distal portion of the colon and the tumor number did not differ between both groups (Fig 2C). However, average tumor size was significantly decreased in MDR1A KO versus WT mice (Fig 2C), with tumors ≤ 3 mm in size predominantly found in MDR1A KO mice.

**Fig 3:**
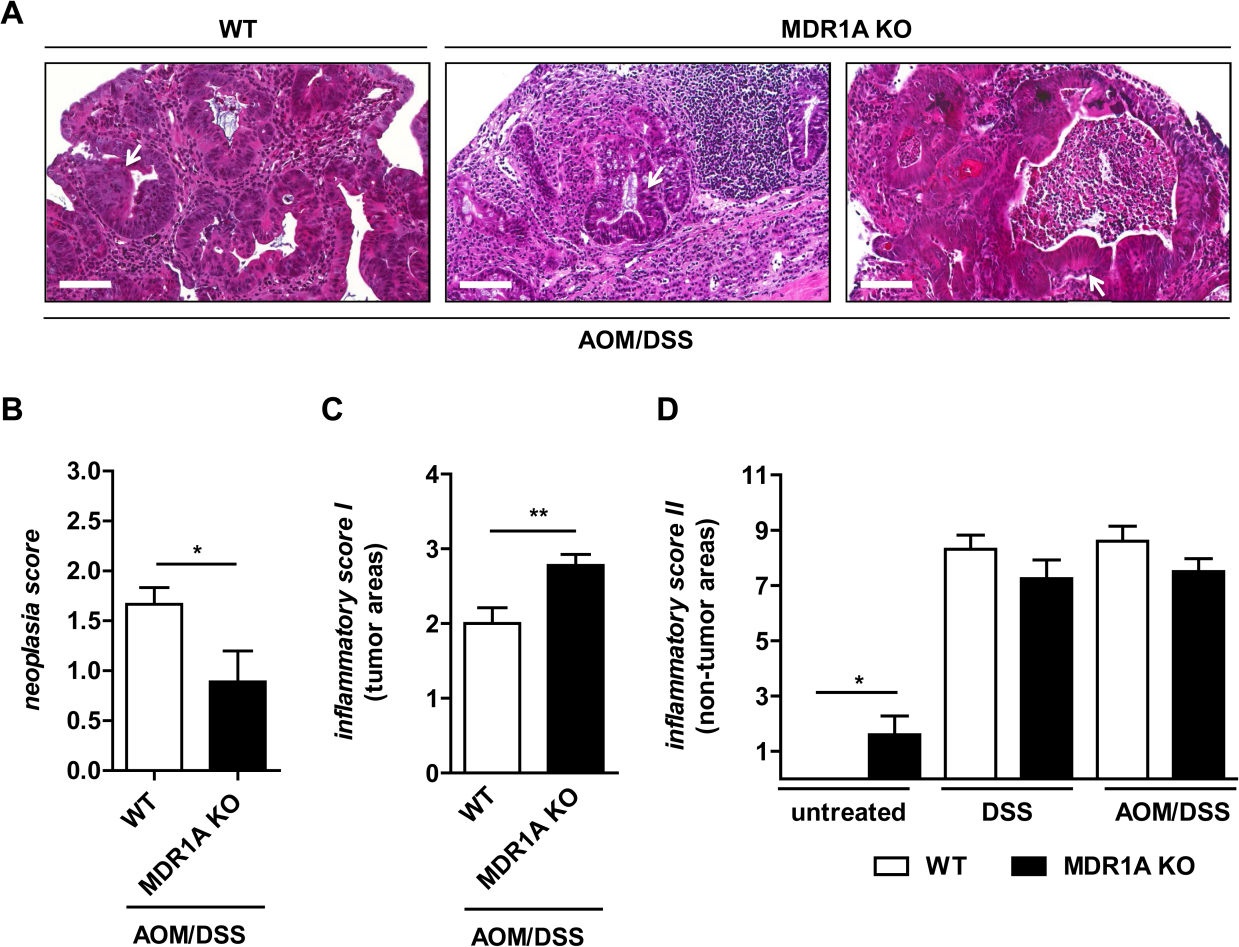
Colonic tumor lesions in MDR1A KO mice show severe inflammation but lower grade dysplasia. (A) Histopathology (H&E) of colonic tumor disease induced by CAC protocol (n≥9/ genotype). Representative sections of colons from WT or MDR1A KO mice treated with AOM/DSS shown from left to right: 1. high-grade dysplastic adenoma (WT); 2. fulminant inflammation with pronounced inflammatory cell infiltrates and reactive epithelial changes, but no dysplasia (MDR1A KO); and 3. adenoma with massive inflammatory infiltrates and moderate epithelial changes consistent with low-grade dysplasia (MDR1A KO). Scale bar: 100μm. *White arrows* indicate exemplary areas of interests. Neoplasia (B) and inflammatory scores for (C) tumor and (D) non-tumor areas of WT vs. MDR1A KO mice (untreated (*n* = 5-6/genotype), DSS (*n* = 8/genotype) or AOM/DSS (*n* = 9-10/genotype)), as described in *Materials and Methods*. Data represent means ± SEM. *p < 0.05, **p < 0.01.

Histological examination (Fig 3A) revealed a trend toward decreased epithelial gland disorganization and fewer cribriform structures consistent with more features of lower grade dysplasia in MDR1A KO tumors than WT tumors. This observation was supported by the neoplasia score (Fig 3B); invasive growth or adenocarcinoma could not be detected. In parallel, MDR1A KO tumors showed increased inflammatory activity compared to WT tumors (Fig 3A and 3C), while the degree of chronic inflammation in non-tumor, mucosal areas was similar between both genotypes after DSS treatment (Fig 3D).

### MDR1A KO mice have increased numbers of tumor-associated B cells and plasma cells

**Fig 4:**
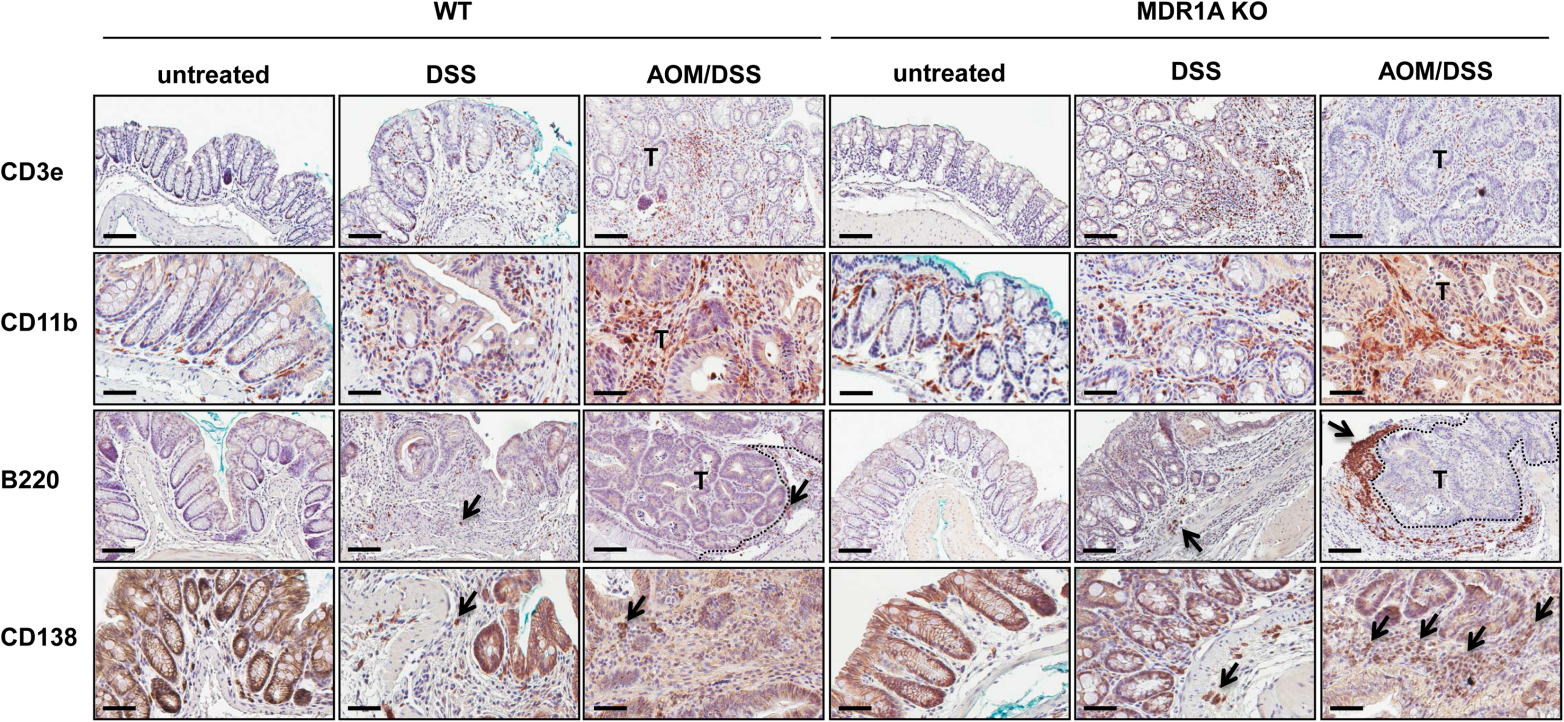
MDR1A KO mice exhibit increased numbers of B cells and plasma cells in the inflamed colonic tumor microenvironment. Representative anti-CD3e, anti-CD11b, anti-B220 and anti-CD138 immunohistochemistry with DAB chromogen and hematoxylin counterstain of distal colons (untreated, DSS, AOM/DSS) from WT and MDR1A KO mice (*n* = 3-9/group). *Black arrows* indicate examples of positive cells. *T* indicates the tumor area; its margin is marked by a dotted line. Scale bars: 100μm (CD3e, B220); 50μm (CD11b, CD138).

Next, we characterized the predominant cell type of the inflammatory infiltrates in MDR1A KO tumors by immunohistochemical staining (Fig 4). No substantial differences in the extent of increased numbers of CD3e+ T lymphocyte cells and CD11b+ myeloid could be detected between the WT and MDR1A KO tumors. However, abundant B220+ B lymphocyte aggregates were recruited to the peritumoral stroma in MDR1A deficiency, while just a few scattered B220+ B lymphocytes were present in the close neighborhood of WT tumors. Microclusters of CD138+ plasma cells infiltrated MDR1A KO tumors, while only few individual CD138+ plasma cells were evident in WT tumor tissues. In contrast, only sparse numbers of B220+ and CD138+ cells were found in the inflamed intestinal mucosa of DSS-MDR1A KO or DSS-WT controls and the extent did not differ between both groups. In addition, no increased frequencies of B220+ B lymphocytes and CD138+ plasma cells were detected by immunohistochemistry in the colonic mucosa of untreated MDR1A KO or WT mice. The general distribution of immature and mature B cells [40, 41] in bone marrow, spleen and colonic lamina propria was comparable between untreated WT and MDR1A KO mice (S2 Fig), suggesting normal B cell development and migration.

### MDR1A-deficient tumors demonstrate enhanced cell injury, leading to impaired spheroid growth ex-vivo

**Fig 5:**
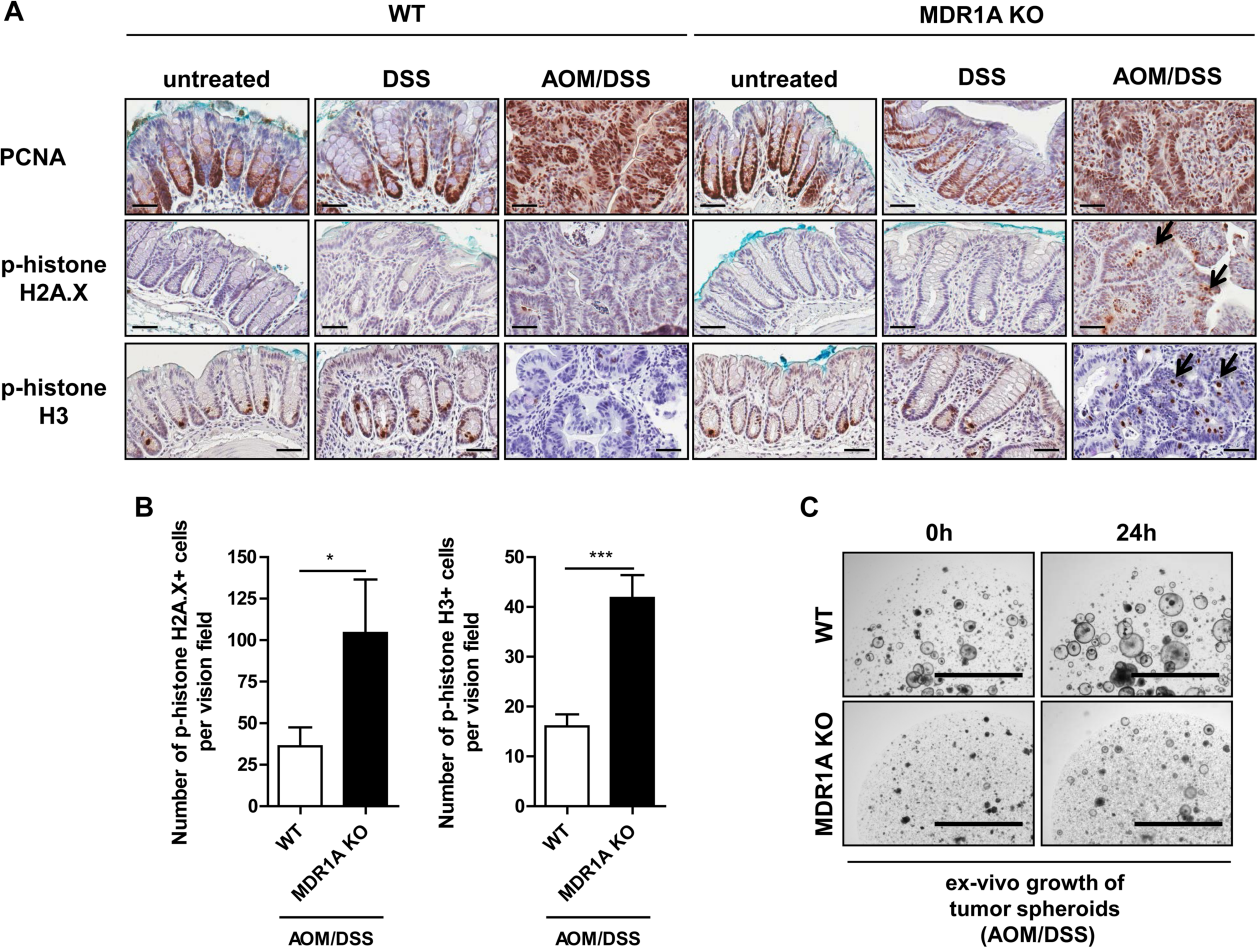
Colonic MDR1A KO tumors show increased cellular damage in-vivo, which correlates with reduced stem cell-enriched spheroid growth ex-vivo. (A) Representative anti-PCNA, anti-p-histone H2A.X and anti-p-histone H3 immunohistochemistry with DAB chromogen and hematoxylin counterstain of distal colons (untreated, DSS) and tumors (AOM/DSS) from WT and MDR1A KO mice (*n* = 3-9/group). Scale bar: 50μm. *Black arrows* indicate examples of positively labelled cells (B) which were quantified by counting at least 5–6 randomly chosen vision fields of each slide at an original magnification 40x (p-histone H2A.X) or 20x (p-histone H3). Data are presented as means ± SEM (*n* = 4–6 mice/group). *p < 0.05; ***p < 0.001. (C) Tumor spheroids from WT or MDR1A KO mice after AOM/DSS treatment were passaged and maintained in Matrigel® in a 6-well plate. On the next day and then 24h later, spheroids were imaged on the EVOS fl (X2) using standardized settings. Shown are representative images of ex-vivo tumor spheroid formations from WT and MDR1A KO after AOM/DSS treatment. Scale bar: 2000μm.

We then examined cell proliferation and injury in WT and MDR1A-deficient tumors by immunohistochemistry. Nuclear staining of PCNA, a marker of cell proliferation [42], was equally increased in WT and MDR1A KO adenomas (Fig 5A), when compared to control tissues. However, analysis of phosphorylated histone H2A.X (p-H2A.X), a marker of genotoxic stress-induced apoptotic DNA fragmentation [43], revealed a significant difference between WT and MDR1A-deficient tumors (Fig 5A and 5B). The average yield of p-H2A.X-positive cells was higher in MDR1A KO tumors compared to WT tumors. P-H2A.X-positive cells were mostly found in the epithelial compartment of MDR1A KO tumors. Furthermore, phosphorylation of histone H3 on Ser-10 (p-histone H3), which may occur when cells are exposed to death stimuli [44], was also enhanced in MDR1A KO tumors (Fig 5A and 5B). We isolated intestinal tumors from AOM/DSS-treated WT and MDR1A KO mice and cultured them short-term ex-vivo as stem cell-enriched spheroids [33]. As shown in Fig 5C, MDR1A KO tumor spheroids grew and expanded markedly slower compared to WT tumor spheroids.

AOM represents a potent DNA damage-inducing agent that mediates p53-dependent intestinal epithelial apoptosis [45]. WT or MDR1A KO mice treated with AOM as a single modality did not develop tumors up to week 12 (data not shown). Furthermore, AOM alone did not induce DNA damage (as assessed by p-H2A.X-positive cells) in MDR1A KO colons (S3 Fig), suggesting that decreased tumorigenesis is not due to altered AOM sensitivity of the colonic epithelium in MDR1A KO mice.

### MDR1A-deficient tumors express high levels of immunoglobulin light chain genes and show altered expression of genes involved in apoptosis and injury

**Fig 6:**
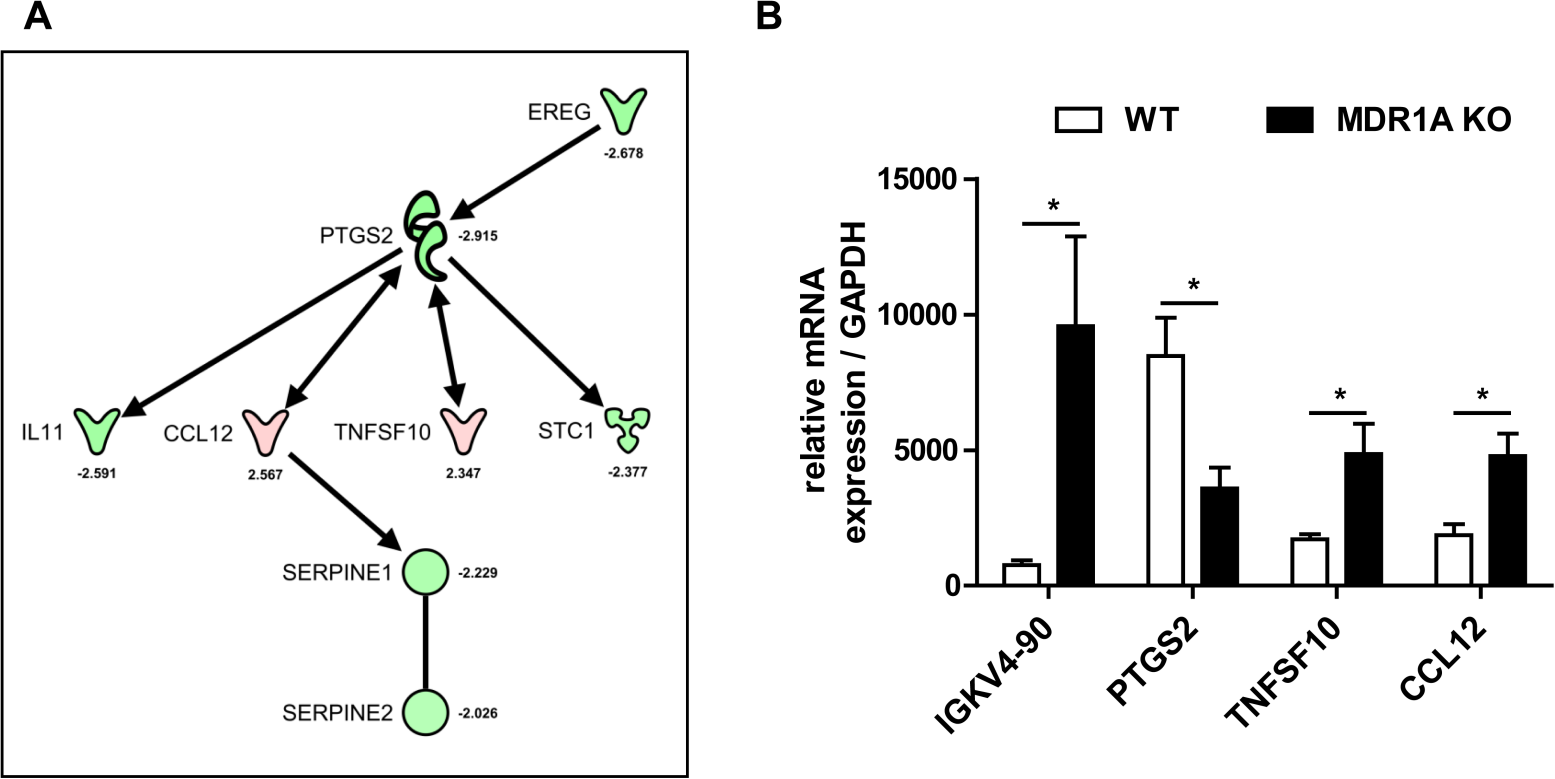
MDR1A-deficient colonic tumors show differentially regulated expression of distinct genes related to B cell activation and inhibition of tumor progression. (A) Molecules identified by microarray analysis to be differentially regulated in MDR1A KO vs. WT tumors were connected into a hypothetical common signaling model by the IPA knowledge base. Genes are shown in a red-to-green color scale, corresponding to their relative abundance in tumors from AOM/DSS-MDR1A KO mice compared to AOM/DSS-WT mice (*red* = higher; *green* = lower). A detailed list of all identified genes with expression levels is provided in S4 Table. (B) Relative expression of selected genes that were differentially regulated in MDR1A KO vs. WT tumors (*n* = 4/genotype), as determined by realtime qPCR analysis. Results are shown in relation to mRNA expression for the housekeeping gene GAPDH. Data are presented as means ± SEM (*n* = 4 individual tumors/genotype, *p < 0.05).

To gain further insight into the underlying host mechanisms responsible for reduced colitis-associated tumor growth in the context of MDR1A deficiency, we performed a broad gene expression profiling analysis to identify potential target genes. We compared mRNA expression levels in tumor tissues between AOM/DSS-exposed WT vs. MDR1A KO mice and identified 57 genes differentially regulated (*p* < 0.05; ±2-fold), which are summarized in S4 Table. Within this set, 16 genes alone belonged to *κ light chain and heavy chain Ig genes* which were significantly upregulated in MDR1A KO tumors, correlating with the enhanced presence of tumor-associated B cells and plasma cells in MDR1A deficiency (Fig 4). As annotated by the IPA Ingenuity knowledge base, 13 additional genes that were significantly altered in MDR1A KO tumors were associated with *apoptosis*, 19 with *organismal injury and abnormalities/cancer* and 9 with *inflammatory responses* as main biologic functions. As shown in Fig 6A, IPA indicated signaling of 7 molecules (CCL12, EREG, IL-11, SERPINE1, SERPINE2, STC1, TNFSF10) to be interconnected via PTGS2 (COX-2). Importantly, PTGS2 was markedly downregulated in MDR1A-deficient tumors compared to WT tumors. Similarly, gene expressions of EREG, a ligand of EGFR, and IL-11 were decreased in MDR1A KO tumors, while gene expressions of CCL12 (MCP-5) and TNFSF10 (TRAIL) were significantly increased in MDR1A KO tumors. Realtime qPCR analysis of this selection of representative genes (IGKV4-90, PTGS2, TNFSF10, CCL12) validated the gene expression changes observed by the array analysis (Fig 6B).

### Absence of adaptive immunity allows tumor progression of murine CAC in MDR1A deficiency

To determine the functional role of the adaptive immune system in our model in-vivo, we bred MDR1A KO mice to immunocompromised RAG2 KO mice to generate MDR1A/RAG2 dKO which lack mature B and T cells. RAG2 KO and MDR1A/RAG2 dKO mice were subjected to the AOM/DSS protocol and followed up to week 20, as initial results suggested delayed overall tumor development in both RAG2-deficient mice strains (data not shown). 50% (7 of 14) of the AOM/DSS-MDR1A/RAG2 dKO mice did not reach the experimental endpoint: 4 mice had to be sacrificed because of body weight loss > 20% (days 11, 30 (twice) and 84) and 3 mice died unexpectedly (days 33, 56 and 58). 10% (1 of 10) of the AOM/DSS-RAG2 KO mice did not reach the experimental endpoint and had to be sacrificed due to body weight loss > 20% (day 54).

**Fig 7:**
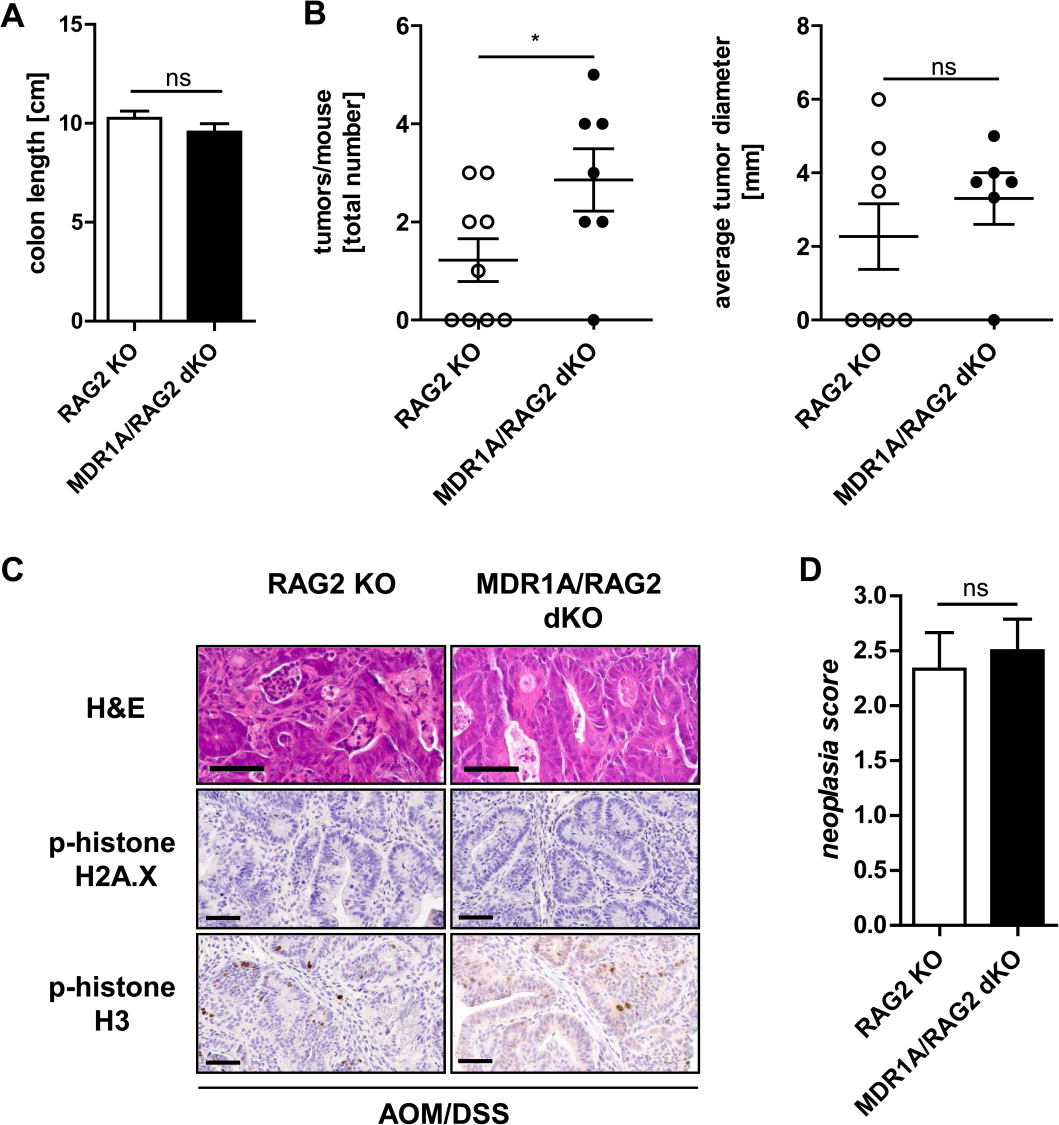
Absence of adaptive immunity decreases tumor cell damage in MDR1A deficiency. RAG2 KO and MDR1A/RAG2 dKO mice were intraperitoneally injected with AOM (10mg/kg body weight) and subjected to 3 cycles of DSS treatment (1 cycle representing 7 days of 2.5% DSS followed by 14 days of H_2_O). In week 20, (A) colon length was assessed and (B) macroscopic tumors in colons were counted and measured. Data pooled from at least two independent experiments are shown (*n* = 7–9 mice/genotype). (C) Representative H&E histopathology, anti-p-histone H2A.X and anti-p-histone H3 immunohistochemistry with DAB chromogen and hematoxylin counterstain (*n* = 6-8/group) of tumors from AOM/DSS-treated RAG2 KO and MDR1A/RAG2 dKO mice. Scale bars: 60μm. (D) Neoplasia score (*n* = 3–4 mice/genotype). (A,B,D) Data are presented as means ± SEM. *p < 0.05, ns: not significant.

Colon length, a marker of inflammation [39], was not reduced in AOM/DSS-treated RAG2 KO and MDR1A/RAG2 dKO mice (Fig 7A) and comparable to untreated, implying that inflammatory activity was not increased. Although the average number of tumors that developed per mouse was generally decreased in RAG2-deficient strains (Fig 7B), compared to WT or MDR1A KO mice (Fig 2C), the remaining MDR1A/RAG2 dKO mice showed a higher tumor burden than RAG2 KO (Fig 7B). However, we found no significant differences in tumor size or histopathology between RAG2 KO and MDR1A/RAG2 dKO (Fig 7B, 7C and 7D); all tumor lesions were either adenomas with high-grade dysplasia or non-invasive adenocarcinoma. Of note, the neoplasia score was significantly higher (*p* < 0.01) in MDR1A/RAG2 dKO tumors (Fig 7D) compared to MDR1A KO tumors (Fig 3B). In contrast to MDR1A KO tumors (Fig 5A and 5B), MDR1A/RAG2 dKO tumors showed hardly any DNA damage, as evidenced by very few p-histone H2A.X-positive cells and only some p-histone H3-positive cells (Fig 7D), which was comparable to RAG2 KO or WT tumors (Fig 5A and 5B). These findings imply that the adaptive arm of the immune system is essential to suppress intestinal tumor growth of murine CAC through enhanced cell injury in MDR1A deficiency.

### MDR1A KO B lymphocytes limit intestinal tumor growth ex-vivo

**Fig 8:**
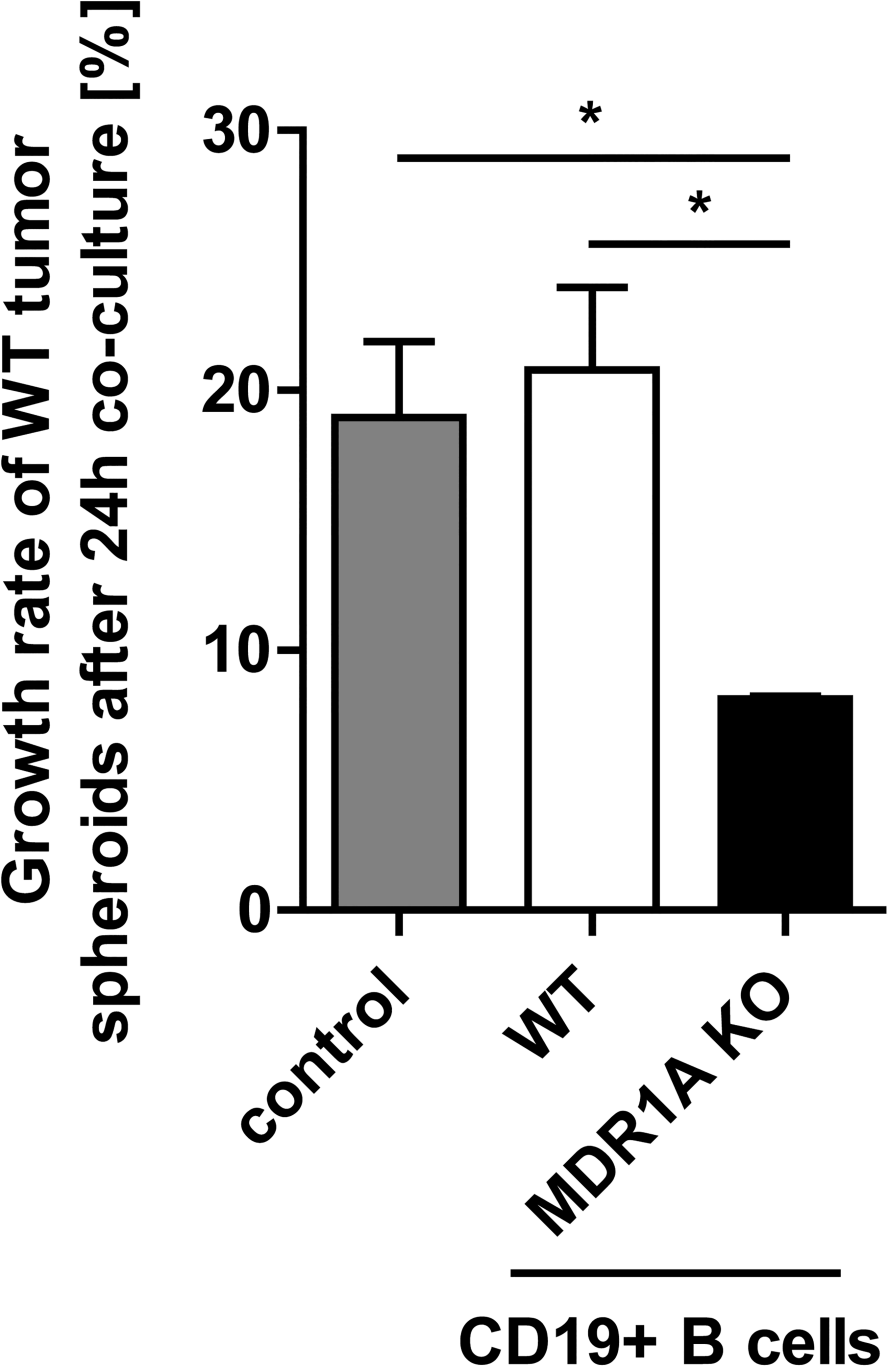
MDR1A-deficient B lymphocytes limit intestinal tumor spheroid growth ex-vivo. As depicted in S1 Fig and described in *Materials and Methods*, WT tumor spheroids (each clone in duplicate; one clone/WT mouse after AOM/DSS treatment) were grown ex-vivo in Matrigel® for 44h in a 6-well plate and then co-cultured for 24h with purified CD19+ B cells (4x10^6^/2ml on a 0.4μm-insert membrane; in duplicate) from untreated, age- and gender-matched WT or MDR1A KO mice. Control only contained full media supplemented with BAFF, but no B cells. Spheroids were imaged on the EVOS fl (X2) before (0h) and after (24h) addition of B cells. Spheroid areas [mm^2^] were measured by ImageJ (applying a known image scale as calibration) and the relative 24h-growth rates were calculated. Results of a representative experiment are shown (*n* = 2). Data are presented as means ± SEM (*n* = 3 tumor spheroid clones, *p < 0.05).

Based on our observations of 1. predominant B cell accumulation in the tumor microenvironment of MDR1A KO mice and 2. marked tumor progression of MDR1A/RAG2 dKO which lack B (and T) cells, we asked next what functional role B cells may play in colon tumor development in MDR1A deficiency. To avoid the high degree of mortality seen in AOM/DSS-MDR1A/RAG2 dKO mice, we established a novel, ex-vivo model, as described in *Materials and Methods* (S1 Fig). Growth of AOM/DSS colonic tumor-derived spheroids from MDR1A KO mice, but not WT, was delayed (Fig 5C) due to increased cell damage (Fig 5B). We therefore co-cultured CD19+ B cells from MDR1A KO mice with AOM/DSS colonic tumor-derived spheroids from WT mice as “tumor target” for 24h in a Transwell® system in a proof-of-concept approach. When WT tumor spheroids were exposed to CD19+ B cells from WT mice, no change in intestinal tumor growth rate was observed (Fig 8) compared to control media alone. However, when WT tumor spheroids were exposed to CD19+ B cells from MDR1A KO mice, this treatment significantly reduced WT tumor growth (Fig 8). These data suggest that MDR1A-deficient B cells may elicit specific anti-tumor immune responses in the microenvironment which contribute to inhibition of intestinal tumor promotion.

## Discussion

Reduced ABCB1/MDR1 mRNA expression has been associated with active UC [46] and early CRC [14]. Loss of MDR1A gene function drives chronic colitis in mice [16, 17, 47]. The present study implies a previously unappreciated role of ABCB1/MDR1 in colonic tumor advancement under chronic inflammatory conditions. We found that lack of ABCB1/MDR1-mediated signals promoted a B cell-dominated tumor immunogenic microenvironment and resulted in an increase in tumor cell damage, which correlated with inhibition of inflammation-associated tumor growth.

First, we showed that the expression pattern of ABCB1/MDR1 differs between human CAC vs. CRC specimens. We confirmed previous observations [14] that ABCB1 mRNA levels are lower in CRC tumor tissues when compared to morphologically normal tumor margin tissues. In contrast, ABCB1 mRNA levels were highly variable in CAC tumor and matched non-tumor tissues. No significant difference was detectable to active UC without colon cancer, but the observed divergence of mRNA levels did not correlate directly with changes in protein abundance. Most tissue samples from active UC – regardless whether with or without colon cancer – exhibited diminished protein expression of apical ABCB1/MDR1 p-gp in IEC of inflamed colons, in contrast to CRC or normal controls. Future studies will need to identify the reasons for these poor correlations between the level of ABCB1/MDR1 mRNA and the level of p-gp protein in human CAC. In general, protein turnover of p-gp appears to be a cell-cycle-dependent process and may occur independently from mRNA regulation, as observed in other cancer cell types [48]. Several signaling pathways, which are also involved in IBD pathogenesis, have been shown to influence protein stability of p-gp, regulating its expression and activity [9, 49] without affecting ABCB1/MDR1 mRNA levels, by e.g. posttranscriptional modifications [50]. Alterations of the ubiquitin-proteasome system in IBD [51] may degrade p-glycoprotein [52]. Pro-inflammatory cytokines in IBD, such as TNFα [53], IL-1ß or IL-6 [54], may secondarily modulate expression and activity of ABCB1/MDR1. In addition, numerous microRNAs, which have been linked to IBD [55], may control p-gp signaling.

Second, we assessed the consequences of ABCB1/MDR1 loss-of-function on CAC in mice. Our results indicate that MDR1A deficiency enhanced intratumoral inflammation and cellular damage, which were associated with reduced colonic tumor size and decreased degree of dysplasia. MDR1A deficiency shifted the enhanced intrinsic inflammatory response towards an anti-tumorigenic microenvironment by targeting the expression of important genes known to be critical modulators of colon cancer progression. We used the global MDR1A gene knockout model [15], which lacks tissue and cell specificity. ABCB1/MDR1 p-gp is expressed in most tissues throughout the organism and by many different cell types of the intestinal mucosal barrier, including intestinal epithelial and immune cells, such as B lymphocytes [56]. Our results reveal that ABCB1/MDR1 loss-of-function affected (at least) these two cell compartments in murine CAC.

Blockade of PTGS2 (COX-2) activity, a key player in colorectal carcinogenesis, inhibits intestinal tumorigenesis [34, 57] and exacerbates inflammation-associated colonic injury [58]. Induction of the PTGS2 pathway increases p-gp activity in-vitro [59]. We show that restrained colonic tumor growth and collateral tissue damage in MDR1A deficiency were associated with marked gene suppression of PTGS2 within intestinal tumors. IL-11, a downstream target of PTGS2 [60] and a potent driver of tumor progression in CAC [61], was significantly reduced in inflamed MDR1A KO tumors compared to WT tumors. Activation of the EGFR signal transduction pathway controls a variety of cellular processes essential for colon cancer progression, including tumor cell survival, proliferation and motility [62]. Both, PTGS2 [63] and ABCB1/MDR1 [64], are regulated by EGFR signaling. We demonstrate that downregulation of PTGS2 coincided with decreased gene expression of the EGFR ligand EREG in inflamed MDR1A-deficient tumors. Thus, lack of ABCB1/MDR1-mediated signals appears to be closely intertwined with concordant suppression of the PTGS2 and EGFR signaling pathways in colitis-associated tumorigenesis, implying severe impairment of epithelial proliferation during inflammatory injury in-vivo [65, 66]. Future studies will need to determine whether signaling of EGFR and PTGS2 may be regulated by a feedback loop mediated through ABCB1/MDR1 – possibly via Platelet-activating factor (PAF), a p-gp substrate [67], which affects EGFR phosphorylation [68] and PTGS2-mediated prostaglandin release [69]. It remains to be examined whether MDR1A deficiency impairs TCF4/ß-catenin responsive elements in the promoter in chronic inflammation, potentially altering the Wnt/ß-catenin signaling pathway [70], which itself interrelates with PTGS2 and EGFR signaling in multidirectional loops [71, 72]. Bystander effects exhibited by MDR1A-deficient inflammatory cells infiltrating the microenvironment as a result of signals received from the nearby tumor cells (and vice versa) remain to be dissected.

Endogenous TNFSF10 (TRAIL), a member of the TNF family of ligands, selectively induces apoptosis of a variety of tumor cells without harming normal, non-transformed cells [73]. Inhibition of PTGS2 or EGFR signaling is known to sensitize tumor cells to TNFSF10-mediated apoptosis [74, 75]. We found enhanced expression of TNFSF10 in inflamed colonic tumors in the context of MDR1A deficiency, associated with accumulation of phosphorylated histone H2A.X and phosphorylated histone H3, as evidence of enhanced DNA damage and cell death [43, 44]. Growth was significantly impaired in MDR1A-deficient colonic tumor spheroids when cultured ex-vivo, suggesting that increased intratumoral damage in MDR1A deficiency affects tumor-propagating cells. An inverse relationship between p-gp and TNFSF10 has previously been described in-vitro using ABCB1/MDR1-transfected HeLa cells [76], however the underlying signaling mechanisms of this correlation and cellular source remain to be studied.

A unique feature of colonic tumors of AOM/DSS-exposed MDR1A KO mice were the large peritumor aggregates of B220+ B cells and interspersed CD138+ plasma cells. Microarray analysis revealed upregulated expression of fragments of predominantly variable regions of Ig light chains within MDR1A KO tumors, likely representing tumor-reactive and/or auto-reactive antibodies. Inflamed MDR1A KO tumors showed increased expression of the CCR2 ligand CCL12 (MCP-5) in the microenvironment, which is known to be expressed by recruited inflammatory macrophages [77, 78], specifically attracting B cells [79]. Chronic mucosal inflammation [80] and colorectal tumorigenesis [81] have been associated with altered B cell function, but the exact contribution of B cells and plasma cells in the pathogenesis of CAC (and IBD) remains unknown. MDR1A deficiency protected against tumor progression by accelerating intratumoral cell death, which was dependent on the adaptive immune system. Functionally, we show that MDR1A KO B cells may contribute to intestinal tumor growth inhibition by exerting anti-tumor effects. When co-cultured in close proximity to WT tumor spheroids, MDR1A KO CD19+ B cells, but not WT CD19+ B cells, repressed tumor growth. To enhance B cell survival, our experiments were performed in the presence of BAFF. We cannot exclude that BAFF may have modulated the anti-tumor activity of B lymphocytes in MDR1A deficiency ex-vivo. Future studies will need to identify the specific B cell subset-derived immune responses against CAC in MDR1A deficiency, which may include several tumoricidal mechanisms, such as production of granzyme B [82] and anti-tumor antibodies [83]. It is possible that colonic MDR1A KO B cells represent the cellular source of enhanced cytotoxic TNFSF10 in the inflamed tumor microenvironment [84].

Colon cancer has been linked to alterations in the gut microbiome [5]. Colonic inflammation in MDR1A KO mice is commensal microbiota-dependent [16, 17, 47]. Future studies will also need to investigate how the gut microbiota may affect tumor development and regression in the context of MDR1A deficiency. Changes in commensal composition in MDR1A deficiency, potentially altering host mucosal immune responses to a predominantly pro-apoptotic and anti-proliferative phenotype, remain to be identified. It must be clarified whether bacterial ligands may directly trigger enhanced tumor cell damage and death in MDR1A deficiency. It is possible that bacterial ligands may recruit and render B cells to acquire cytotoxic activities against tumor cells [84] in MDR1A deficiency.

In conclusion, our study establishes a pro-tumor function of ABCB1/MDR1 in murine CAC. Loss of ABCB1/MDR1 protein function in human UC may represent an important intrinsic host defense mechanism to attenuate tumor progression of CAC.

## Supporting information

S1 Table List of antibodies. (PDF)

S2 Table Histopathologic patient characteristics. (PDF)

S3 Table Conditions of immunohistochemistry. (PDF)

S4 Table Gene expression profiling by microarray analysis of MDR1A KO vs. WT tumors induced by AOM/DSS. Genes significantly regulated (AOM/DSS-MDR1A KO tumors vs. AOM/DSS-WT tumors) were included, as described in *Materials and Methods*. *P* values <0.05 are marked in yellow; >2-fold changes in red (upregulated) or blue (downregulated), respectively. Gene accession numbers, gene symbols, gene descriptions, Gene Ontology (GO) annotations (molecular functions and biological processes) and IPA associations are listed for each specific gene. The identified κ light chain and heavy chain Ig genes are shaded in grey.
(PDF)

S1 Fig Schematic representation of the co-culture experiments of either WT CD19+ B cells or MDR1A KO CD19+ B cells or control media (no B cells) with AOM/DSS-tumor derived WT spheroids. (TIF)

S2 Fig Relative frequencies of different B cell subsets. The relative frequencies of different B cell subsets (*pro-B*: B220+CD43+; *pre-B*: B220+CD43-IgD-IgM-; *immature B*: B220+CD43-IgD-IgM+; *mature B*: B220+CD43-IgD+IgM+; *plasma cells*: B220-CD19-CXCR4+CD138+) in (A) bone marrow, (B) spleen and (C) colonic lamina propria from untreated WT and MDR1A KO (~23 wk old; *n* = 3/group) were determined by flow cytometry (as percentage of all live cells). Results show means ± SEM of 3 independent experiments.
(TIF)

S3 Fig No evidence of increased intestinal epithelial cell damage after AOM treatment of MDR1A KO mice. Representative immunofluorescent staining with anti-p-histone H2A.X (FITC, green) of distal colons from untreated vs. AOM-treated MDR1A KO mice (*n* **=** 3-4/group), as assessed by confocal laser microscopy. Scale bar: 100μm. Nuclei were counterstained with propidium iodide (red).
(TIF)
