# Supplementary material for: MDR1A deficiency restrains tumor growth in murine colitis-associated carcinogenesis
Source: PLoS One. 2017 Jul 7;12(7):e0180834. doi: 10.1371/journal.pone.0180834 (PMC5501609; doi:10.1371/journal.pone.0180834)
Supplement: S1 Table — (PDF) [file pone.0180834.s001.pdf]

**Antibody**	**Company**	**Product no.**	**Clone**	**Isotype**
Immunhistochemistry & Immunofluorescence				
CD3	Thermo Fisher	MA5-14524	SP7	Rabbit IgG
CD11b	abcam	ab133357	EPR1344	Rabbit IgG
CD45R/B220	eBioscience	14-0452	RA3-6B2	Rat IgG2a, kappa
CD138/Syndekan-1	Biolegend	142501	281-2	Rat IgG2a, kappa
E-Cadherin	Cell Signaling	3195		Rabbit IgG
MDR1/ABCB1	Cell Signaling	13978	E1Y7S	Rabbit IgG
Non-phospho (Active) beta-Catenin (Ser33/37/Thr41)	Cell Signaling	8814	D13A1	Rabbit IgG
PCNA	Cell Signaling	13110	D3H8P	Rabbit IgG
Phospho-Histone H2A.X (Ser139)	Cell Signaling	9718	20E3	Rabbit IgG
Phospho-Histone H3 (Ser10)	Cell Signaling	9701		Rabbit IgG
FACS				
APC Anti-Mouse CD43	BD Pharmingen™	560663	S7	Rat IgG2a, kappa
APC Anti-Mouse CD138/Syndecan-1	Biolegend	142505	281-2	Rat IgG2a, kappa
FITC Anti-Mouse IgM	eBioscience	11-5790-81	II/41	Rat IgG2a, kappa
PE Anti-Mouse CD184/CXCR4	Biolegend	146505	L276F12	Rat IgG2b, kappa
PE Anti-Mouse IgD	BD Pharmingen™	558597	11-26c.2a	Rat IgG2a, kappa
PE-Cy™7 Anti-Mouse CD45R/B220	BD Pharmingen™	552772	RA3-6B2	Rat IgG2a, kappa
PerCP-Cy™5.5 Rat Anti-Mouse CD19	BD Pharmingen™	551001	1D3	Rat IgG2a, kappa
