# Supplementary material for: MDR1A deficiency restrains tumor growth in murine colitis-associated carcinogenesis
Source: PLoS One. 2017 Jul 7;12(7):e0180834. doi: 10.1371/journal.pone.0180834 (PMC5501609; doi:10.1371/journal.pone.0180834)
Supplement: S2 Table — (PDF) [file pone.0180834.s002.pdf]

	CRC	CAC	UC
**Total no. of patients**	16	13	25
**Stage (UICC)**			
**I (T1 or T2, N0, M0)**	1 (6%)	6 (47%)	
**II (T3 or T4, N0, M0)**	4 (25%)	3 (23%)	
**III (any T, any N, M0)**	5 (31%)	2 (15%)	
**IV (any T, any N, M1)**	6 (38%)	2 (15%)	
**Active inflammation**	R_0: 0 (0%)	R_0: 13 (100%)	25 (100%)
	Tumor: 16 (100%)	Tumor: 13 (100%)	

CRC: colorectal cancer; CAC: colitis-associated colorectal cancer; UC: Ulcerative Colitis.
