# Supplementary material for: MDR1A deficiency restrains tumor growth in murine colitis-associated carcinogenesis
Source: PLoS One. 2017 Jul 7;12(7):e0180834. doi: 10.1371/journal.pone.0180834 (PMC5501609; doi:10.1371/journal.pone.0180834)
Supplement: S3 Table — (PDF) [file pone.0180834.s003.pdf]

Antibody	antigen retrieval	blocking	dilution	detection reagent	DAB
CD3	Antigen-Retrieval AR-10 (Biogenex, Fremont, CA)	2% BSA/Power Block (Biogenex, Fremont, CA) 10min	1:150 in 1% BSA/TBST 1h RT	SignalStain® Boost IHC Detection Reagent (HRP, Rabbit) (Cell Signaling)	45s
CD11b	Antigen-Retrieval AR-10	2% BSA/Power Block 10min	1:4000 in 1% BSA/TBST 1h RT	SignalStain® Boost IHC Detection Reagent (HRP, Rabbit)	45s
CD45R/B220	10mM Sodium Citrat buffer, pH 6.0	2% BSA/Power Block 10min	1:250 in 1% BSA/TBST 1h RT	ImmPRESS™ HRP Anti-Rat IgG (Vector Laboratories)	30s
CD138/Syndekan-1	10mM Sodium Citrat buffer, pH 6.0	2% BSA/Power Block 10min	1:750 in 1% BSA/TBST 1h RT	ImmPRESS™ HRP Anti-Rat IgG	20s
E-Cadherin	10mM Sodium Citrat buffer, pH 6.0	5% NGS/TBST 1h	1:400 in 5% NGS/TBST o/n 4°C	SignalStain® Boost IHC Detection Reagent (HRP, Rabbit)	30s
MDR1/ABCB1	10mM Sodium Citrat buffer, pH 6.0	5% NGS/TBST 1h	1:250-350 in 5% NGS/TBST o/n 4°C	SignalStain® Boost IHC Detection Reagent (HRP, Rabbit)	5min
Non-phospho (Active) beta-Catenin	10mM Sodium Citrat buffer, pH 6.0	5% NGS/TBST 1h	1:800 in 5% NGS/TBST o/n 4°C	SignalStain® Boost IHC Detection Reagent (HRP, Rabbit)	30s
PCNA	10mM Sodium Citrat buffer, pH 6.0	5% NGS/TBST 1h	1:1000 in 5% NGS/TBST o/n 4°C	SignalStain® Boost IHC Detection Reagent (HRP, Rabbit)	1min
Phospho-Histone H2A.X	10mM Sodium Citrat buffer, pH 6.0	5% NGS/TBST 1h	1:100 in 5% NGS/TBST o/n 4°C	SignalStain® Boost IHC Detection Reagent (HRP, Rabbit)	80s
Phospho-Histone H3	10mM Sodium Citrat buffer, pH 6.0	5% NGS/TBST 1h	1:100 in 5% NGS/TBST o/n 4°C	SignalStain® Boost IHC Detection Reagent (HRP, Rabbit)	5min

[BSA: bovine serum albumin; NGS: normal goat serum; RT: room temperature; TBST: Tris buffered saline with Tween 20; HRP: horseradish peroxidase]
