# Supplementary material for: MDR1A deficiency restrains tumor growth in murine colitis-associated carcinogenesis
Source: PLoS One. 2017 Jul 7;12(7):e0180834. doi: 10.1371/journal.pone.0180834 (PMC5501609; doi:10.1371/journal.pone.0180834)
Supplement: S4 Table — Genes significantly regulated (AOM/DSS-MDR1A KO tumors vs. AOM/DSS-WT tumors) were included, as described in Materials and Methods. P values <0.05 are marked in yellow; >2-fold changes in red (upregulated) or blue (downregulated), respectively. Gene accession numbers, gene symbols, gene descriptions, Gene Ontology (GO) annotations (molecular functions and biological processes) and IPA associations are listed for each specific gene. The identified κ light chain and heavy chain Ig genes are shaded in grey. (PDF) [file pone.0180834.s004.pdf]

Gene Accession	Gene Symbol	Gene Description	Fold Change_KO vs WT	p-value_KO vs WT	GO Annotations - Molecular function	GO Annotations - Biological Process	apoptosis	organismal injury and abnormalities / cancer	inflammatory responses
XR_397994	BC023105	cDNA sequence BC023105	2.39	0.0400					
NM_030728	Cemip	cell migration inducing protein, hyaluronan binding	-2.72	0.0335	clathrin heavy chain binding; hyaluronic acid binding; hyaluronoglucosaminidase activity; hydrolase activity	hyaluronan biosynthetic process; cell migration; release of sequestered calcium ion into cytosol		x	
NM_029586	Cep112	centrosomal protein 112	2.93	0.0042	protein binding	receptor localization to synapse		x	
XR_864385	Cep112os2	centrosomal protein 112, opposite strand 2	2.27	0.0032					
NM_011331	Ccl12	chemokine (C-C motif) ligand 12	2.57	0.0074	CCR chemokine receptor binding; chemokine activity; cytokine activity; inflammatory response		x	x	x
NM_030601	Clca3a2	chloride channel accessory 3A2	2.03	0.0398	intracellular calcium activated chloride channel activity	anoikis; extrinsic apoptotic signaling pathway in absence of ligand	x		
NM_010050	Dio2	deiodinase, iodothyronine, type II	-4.72	0.0074	hormone activity; oxidoreductase activity; thyroxine 5'-deiodinase activity	hormone biosynthetic process; oxidation-reduction process; response to stress; response to lipopolysaccharide		x	
NM_007950	Ereg	epiregulin	-2.68	0.0299	epidermal growth factor receptor binding; growth factor activity	epidermal growth factor receptor signaling pathway; ERBB2 signaling pathway; cell proliferation; wound healing	x	x	x
NR_015554	AI506816	expressed sequence AI506816	-2.13	0.0388					
NM_175473	Fras1	Fraser syndrome 1 homolog (human)	2.05	0.0166	metal ion binding; protein binding	cell communication; morphogenesis of an epithelium		x	
NM_146017	Gabrp	gamma-aminobutyric acid (GABA) A receptor, pi	2.24	0.0403	chloride channel activity; GABA-A receptor activity	chloride transport; signal transduction		x	x
NR_110441	Hottip	Hoxa distal transcript antisense RNA	2.14	0.0010					
OTTMUST00000131410	Ighv1-77	***immunoglobulin heavy variable 1-77***	3.96	0.0500					
AF045503	Ighv1-84	***immunoglobulin heavy variable 1-84***	2.53	0.0476					
OTTMUST00000132406	Igkv13-84	***immunoglobulin kappa chain variable 13-84***	2.18	0.0428					
OTTMUST00000132285	Igkv4-90	***immunoglobulin kappa chain variable 4-90***	9.11	0.0024					
OTTMUST00000133006	Igkv8-30	***immunoglobulin kappa chain variable 8-30***	4.75	0.0009					
OTTMUST00000132847	Igkv12-46	***immunoglobulin kappa variable 12-46***	3.38	0.0287					
OTTMUST00000133377	Igkv3-1	***immunoglobulin kappa variable 3-1***	12.05	0.0081					
OTTMUST00000132841	Igkv4-50	***immunoglobulin kappa variable 4-50***	2.83	0.0410					
OTTMUST00000132745	Igkv4-57	***immunoglobulin kappa variable 4-57***	2.23	0.0470					
OTTMUST00000132705	Igkv4-58	***immunoglobulin kappa variable 4-58***	2.36	0.0111					
OTTMUST00000132641	Igkv4-63	***immunoglobulin kappa variable 4-63***	2.33	0.0191					
M19905	Igkv4-68	***immunoglobulin kappa variable 4-68***	2.12	0.0316					
OTTMUST00000132281	Igkv4-92	***immunoglobulin kappa variable 4-92***	2.38	0.0210					
OTTMUST00000133226	Igkv6-14	***immunoglobulin kappa variable 6-14***	6.07	0.0021					
AF045512	Igkv6-23	***immunoglobulin kappa variable 6-23***	6.17	0.0365					
OTTMUST00000131783	Igkv9-123	***immunoglobulin kappa variable 9-123***	5.10	0.0084					
NM_001025602	Il1rl1	interleukin 1 receptor-like 1	-2.01	0.0350	cytokine receptor activity; interleukin-1 receptor activity; interleukin-33 receptor activity	cytokine-mediated signaling pathway; cell proliferation; inflammatory response; macrophage activation	x	x	x
NM_001290423	Il11	interleukin 11	-2.59	0.0237	cytokine activity; growth factor activity; interleukin-11 receptor binding	cell-cell signaling; cell proliferation; regulation of MAPK cascade	x		x
NM_010663	Krt17	keratin 17	2.10	0.0013	MHC class II receptor activity; protein binding; structural constituent of cytoskeleton; structural molecule activity	morphogenesis of an epithelium; cell growth	x		x
NM_008474	Krt84	keratin 84	3.27	0.0034	structural constituent of cytoskeleton; structural molecule activity	cytoskeleton organization		x	
ENSMUST00000116685	Mir3096	microRNA 3096	-2.61	0.0019					
ENSMUST00000082394	mt-Tq	mitochondrially encoded tRNA glutamine	-2.01	0.0114	triplet codon-amino acid adaptor activity	translation			
NM_021509	Moxd1	monooxygenase, DBH-like 1	2.48	0.0001	catalytic activity; copper ion binding; metal ion binding; monooxygenase activity; oxidoreductase activity; oxidoreductase activity, acting on paired donors, with incorporation or reduction of molecular oxygen, reduced ascorbate as one donor, and incorporation of one atom of oxygen; protein binding	oxidation-reduction process			
NM_172203	Nox1	NADPH oxidase 1	2.09	0.0480	metal ion binding; NADP binding; oxidoreductase activity	angiogenesis; cell migration; inflammatory response; oxidation-reduction process; respiratory burst; response to reactive oxygen species; superoxide anion generation	x		
NM_010933	Nppc	natriuretic peptide type C	-2.25	0.0145	hormone activity; neuropeptide hormone activity	regulation of cell proliferation; response to hypoxia			
NM_178591	Nrg1	neuregulin 1	-2.32	0.0059	chemorepellent activity; cytokine activity; ErbB-2 class receptor binding	cell communication; cell migration; cell proliferation; ERBB2 signaling pathway; wound healing	x		
NM_001005485	Olfr111	olfactory receptor 111	5.49	0.0018	G-protein coupled receptor activity; olfactory receptor activity	detection of chemical stimulus involved in sensory perception of smell; G-protein coupled receptor signaling pathway		x	
NM_146879	Olfr330	olfactory receptor 330	3.42	0.0451	G-protein coupled receptor activity; olfactory receptor activity	detection of chemical stimulus involved in sensory perception of smell; G-protein coupled receptor signaling pathway		x	
NM_008760	Ogn	osteoglycin	2.62	0.0034	growth factor activity; heparin binding; Roundabout binding	keratan sulfate biosynthetic process; regulation of axonogenesis		x	

Gene Accession	Gene Symbol	Gene Description	Fold Change_KO vs WT	p-value_KO vs WT	GO Annotations - Molecular function	GO Annotations - Biological Process	apoptosis	organismal injury and abnormalities / cancer	inflammatory responses
NM_029639	Plet1	placenta expressed transcript 1	2.00	0.0466		cell differentiation; cell-matrix adhesion; cell migration; wound healing			
XR_390704	Gm12602	predicted gene 12602	2.19	0.0426					
ENSMUST00000157288	Gm24915	predicted gene, 24915	2.30	0.0140					
ENSMUST00000158572	Gm25767	predicted gene, 25767	2.42	0.0068					
NM_011198	Ptgs2	prostaglandin-endoperoxide synthase 2	-2.92	0.0187	arachidonate 15-lipoxygenase activity; dioxygenase activity; peroxidase activity; prostaglandin-endoperoxide synthase activity	cellular component movement; cyclooxygenase pathway; inflammatory response; oxidation-reduction process; regulation of apoptotic process; cell proliferation; response to oxidative stress	x		x
NM_009034	Rbp2	retinol binding protein 2, cellular	-2.10	0.0115	lipid binding; retinol binding; transporter activity	retinoid metabolic process; transport			
NM_001289757	Rnf32	ring finger protein 32	2.05	0.0254	metal ion binding; zinc ion binding			x	
NM_011348	Sema3e	sema domain, immunoglobulin domain (Ig), short basic domain, secreted, (semaphorin) 3E	2.01	0.0140	chemorepellent activity; semaphorin receptor binding	angiogenesis; cell differentiation; negative chemotaxis; regulation of actin cytoskeleton reorganization		x	
NM_008871	Serpine1	serine (or cysteine) peptidase inhibitor, clade E, member 1	-2.23	0.0438	serine-type endopeptidase inhibitor activity	angiogenesis; cellular response to ATP; defense response to Gram-negative bacterium; inflammatory response; response to reactive oxygen species; tissue regeneration; wound healing	x	x	x
NM_009255	Serpine2	serine (or cysteine) peptidase inhibitor, clade E, member 2	-2.03	0.0017	serine-type endopeptidase inhibitor activity	cell differentiation; cell growth; wound healing	x		
NM_011371	St6galnac1	ST6 (alpha-N-acetyl-neuraminyl-2,3-beta-galactosyl-1,3)-N-acetylgalactosaminide alpha-2,6-sialyltransferase 1	-5.12	0.0003	alpha-N-acetylgalactosaminide alpha-2,6-sialyltransferase activity; transferase activity	ganglioside biosynthetic process; oligosaccharide biosynthetic process		x	
NM_009285	Stc1	stanniocalcin 1	-2.38	0.0204	hormone activity	cellular response to cAMP; cellular response to hypoxia; cell migration	x	x	
NM_001290728	Sytl5	synaptotagmin-like 5	-2.02	0.0047	calcium-dependent phospholipid binding; clathrin binding; syntaxin binding	regulation of calcium ion-dependent exocytosis; vesicle fusion			
OTTMUST00000134753	Trgj1	T cell receptor gamma joining 1	2.24	0.0305					
NM_022413	Trpv6	transient receptor potential cation channel, subfamily V, member 6	2.39	0.0048	calcium activated cation channel activity; calmodulin binding	calcium ion homeostasis;calcium ion transport		x	
NM_009425	Tnfsf10	tumor necrosis factor (ligand) superfamily, member 10	2.35	0.0169	cytokine activity; tumor necrosis factor receptor superfamily binding	cell-cell signaling; immune response; positive regulation of apoptotic process	x	x	x
