# Supplementary figures and images for: MDR1A deficiency restrains tumor growth in murine colitis-associated carcinogenesis
Source: PLoS One. 2017 Jul 7;12(7):e0180834. doi: 10.1371/journal.pone.0180834 (PMC5501609; doi:10.1371/journal.pone.0180834)

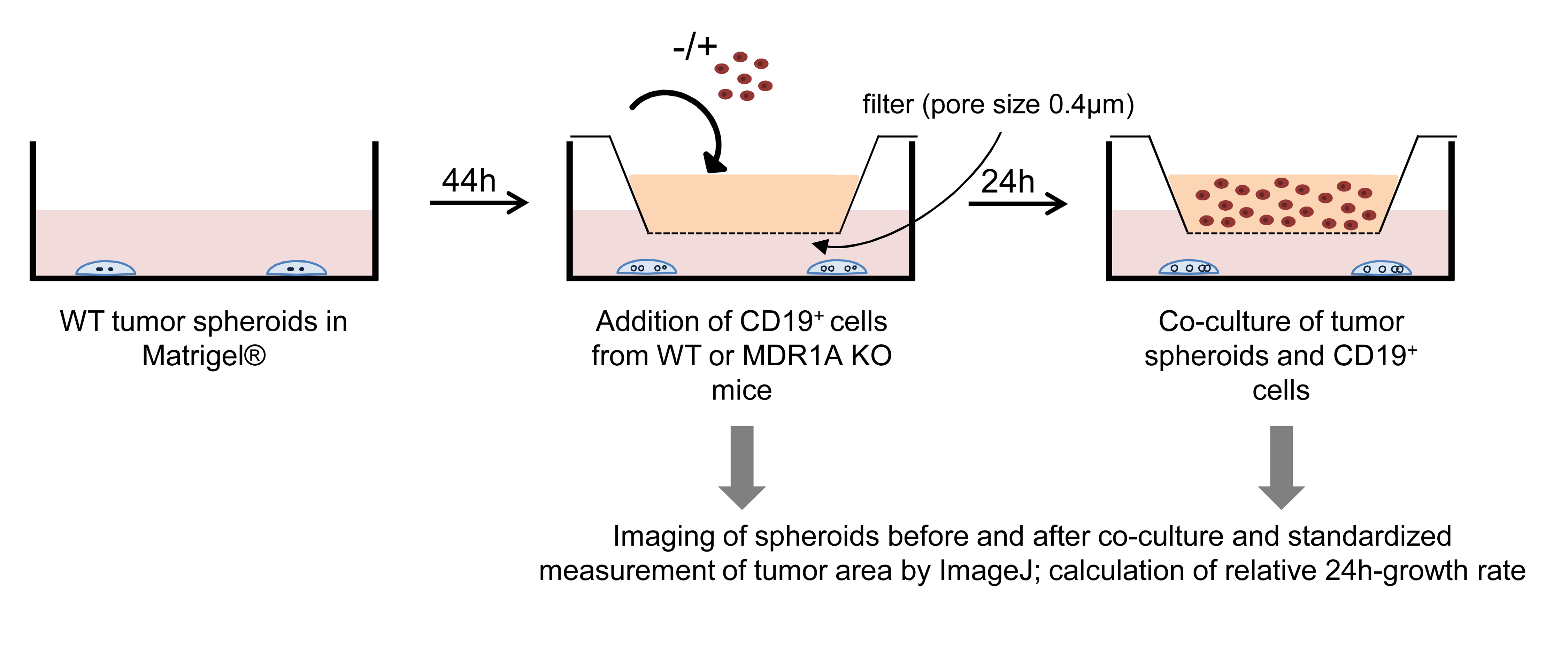

Supplement: S1 Fig — (TIF) [file pone.0180834.s005.tif]

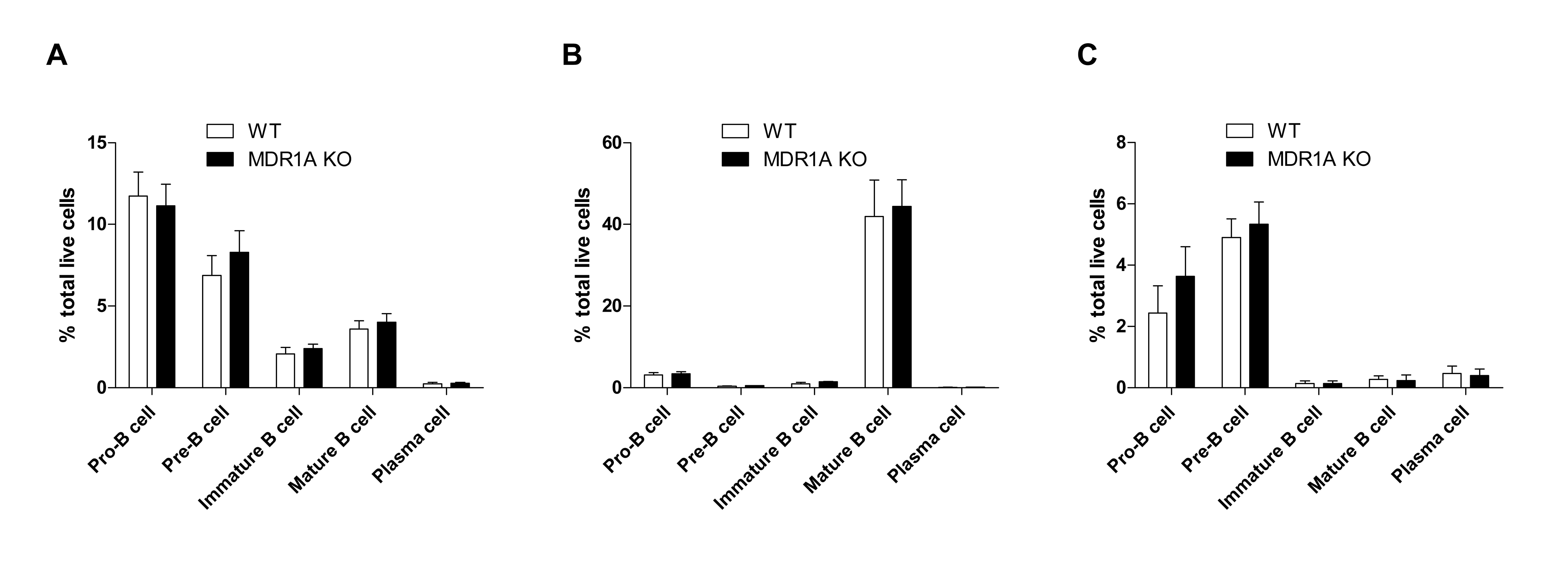

Supplement: S2 Fig — The relative frequencies of different B cell subsets (pro-B: B220+CD43+; pre-B: B220+CD43-IgD-IgM-; immature B: B220+CD43-IgD-IgM+; mature B: B220+CD43-IgD+IgM+; plasma cells: B220-CD19-CXCR4+CD138+) in (A) bone marrow, (B) spleen and (C) colonic lamina propria from untreated WT and MDR1A KO (~23 wk old; n = 3/group) were determined by flow cytometry (as percentage of all live cells). Results show means ± SEM of 3 independent experiments. (TIF) [file pone.0180834.s006.tif]

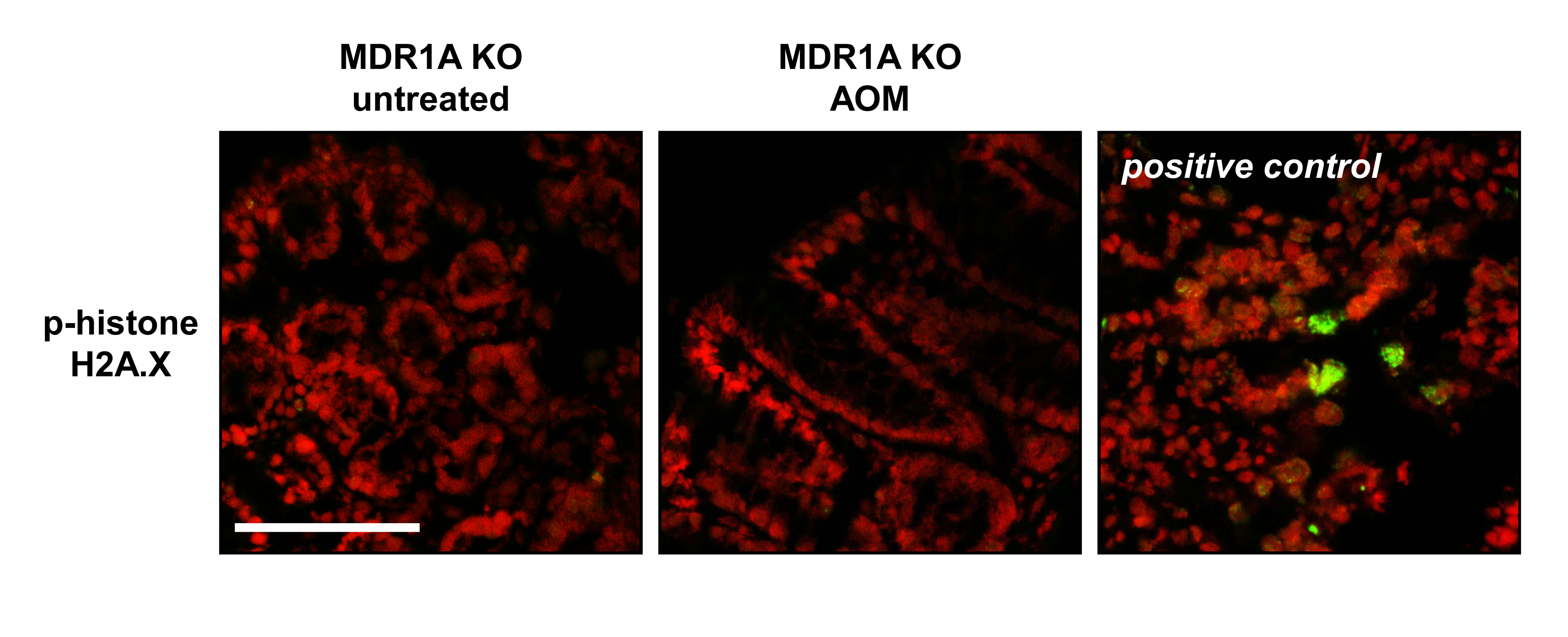

Supplement: S3 Fig — Representative immunofluorescent staining with anti-p-histone H2A.X (FITC, green) of distal colons from untreated vs. AOM-treated MDR1A KO mice (n = 3-4/group), as assessed by confocal laser microscopy. Scale bar: 100μm. Nuclei were counterstained with propidium iodide (red). (TIF) [file pone.0180834.s007.tif]
